# Measurement of differential cross-sections of a single top quark produced in association with a *W* boson at $\sqrt{s}={13}{\text {TeV}}$ with ATLAS

**DOI:** 10.1140/epjc/s10052-018-5649-8

**Published:** 2018-03-06

**Authors:** M. Aaboud, G. Aad, B. Abbott, O. Abdinov, B. Abeloos, S. H. Abidi, O. S. AbouZeid, N. L. Abraham, H. Abramowicz, H. Abreu, Y. Abulaiti, B. S. Acharya, S. Adachi, L. Adamczyk, J. Adelman, M. Adersberger, T. Adye, A. A. Affolder, Y. Afik, C. Agheorghiesei, J. A. Aguilar-Saavedra, S. P. Ahlen, F. Ahmadov, G. Aielli, S. Akatsuka, H. Akerstedt, T. P. A. Åkesson, E. Akilli, A. V. Akimov, G. L. Alberghi, J. Albert, P. Albicocco, M. J. Alconada Verzini, S. C. Alderweireldt, M. Aleksa, I. N. Aleksandrov, C. Alexa, G. Alexander, T. Alexopoulos, M. Alhroob, B. Ali, M. Aliev, G. Alimonti, J. Alison, S. P. Alkire, B. M. M. Allbrooke, B. W. Allen, P. P. Allport, A. Aloisio, A. Alonso, F. Alonso, C. Alpigiani, A. A. Alshehri, M. I. Alstaty, B. Alvarez Gonzalez, D. Álvarez Piqueras, M. G. Alviggi, B. T. Amadio, Y. Amaral Coutinho, C. Amelung, D. Amidei, S. P. Amor Dos Santos, S. Amoroso, C. Anastopoulos, L. S. Ancu, N. Andari, T. Andeen, C. F. Anders, J. K. Anders, K. J. Anderson, A. Andreazza, V. Andrei, S. Angelidakis, I. Angelozzi, A. Angerami, A. V. Anisenkov, N. Anjos, A. Annovi, C. Antel, M. Antonelli, A. Antonov, D. J. Antrim, F. Anulli, M. Aoki, L. Aperio Bella, G. Arabidze, Y. Arai, J. P. Araque, V. Araujo Ferraz, A. T. H. Arce, R. E. Ardell, F. A. Arduh, J-F. Arguin, S. Argyropoulos, M. Arik, A. J. Armbruster, L. J. Armitage, O. Arnaez, H. Arnold, M. Arratia, O. Arslan, A. Artamonov, G. Artoni, S. Artz, S. Asai, N. Asbah, A. Ashkenazi, L. Asquith, K. Assamagan, R. Astalos, M. Atkinson, N. B. Atlay, K. Augsten, G. Avolio, B. Axen, M. K. Ayoub, G. Azuelos, A. E. Baas, M. J. Baca, H. Bachacou, K. Bachas, M. Backes, P. Bagnaia, M. Bahmani, H. Bahrasemani, J. T. Baines, M. Bajic, O. K. Baker, P. J. Bakker, E. M. Baldin, P. Balek, F. Balli, W. K. Balunas, E. Banas, A. Bandyopadhyay, Sw. Banerjee, A. A. E. Bannoura, L. Barak, E. L. Barberio, D. Barberis, M. Barbero, T. Barillari, M-S Barisits, J. T. Barkeloo, T. Barklow, N. Barlow, S. L. Barnes, B. M. Barnett, R. M. Barnett, Z. Barnovska-Blenessy, A. Baroncelli, G. Barone, A. J. Barr, L. Barranco Navarro, F. Barreiro, J. Barreiro Guimarães da Costa, R. Bartoldus, A. E. Barton, P. Bartos, A. Basalaev, A. Bassalat, R. L. Bates, S. J. Batista, J. R. Batley, M. Battaglia, M. Bauce, F. Bauer, K. T. Bauer, H. S. Bawa, J. B. Beacham, M. D. Beattie, T. Beau, P. H. Beauchemin, P. Bechtle, H. P. Beck, H. C. Beck, K. Becker, M. Becker, C. Becot, A. J. Beddall, A. Beddall, V. A. Bednyakov, M. Bedognetti, C. P. Bee, T. A. Beermann, M. Begalli, M. Begel, J. K. Behr, A. S. Bell, G. Bella, L. Bellagamba, A. Bellerive, M. Bellomo, K. Belotskiy, O. Beltramello, N. L. Belyaev, O. Benary, D. Benchekroun, M. Bender, N. Benekos, Y. Benhammou, E. Benhar Noccioli, J. Benitez, D. P. Benjamin, M. Benoit, J. R. Bensinger, S. Bentvelsen, L. Beresford, M. Beretta, D. Berge, E. Bergeaas Kuutmann, N. Berger, L. J. Bergsten, J. Beringer, S. Berlendis, N. R. Bernard, G. Bernardi, C. Bernius, F. U. Bernlochner, T. Berry, P. Berta, C. Bertella, G. Bertoli, I. A. Bertram, C. Bertsche, G. J. Besjes, O. Bessidskaia Bylund, M. Bessner, N. Besson, A. Bethani, S. Bethke, A. Betti, A. J. Bevan, J. Beyer, R. M. Bianchi, O. Biebel, D. Biedermann, R. Bielski, K. Bierwagen, N. V. Biesuz, M. Biglietti, T. R. V. Billoud, H. Bilokon, M. Bindi, A. Bingul, C. Bini, S. Biondi, T. Bisanz, C. Bittrich, D. M. Bjergaard, J. E. Black, K. M. Black, R. E. Blair, T. Blazek, I. Bloch, C. Blocker, A. Blue, U. Blumenschein, S. Blunier, G. J. Bobbink, V. S. Bobrovnikov, S. S. Bocchetta, A. Bocci, C. Bock, M. Boehler, D. Boerner, D. Bogavac, A. G. Bogdanchikov, C. Bohm, V. Boisvert, P. Bokan, T. Bold, A. S. Boldyrev, A. E. Bolz, M. Bomben, M. Bona, M. Boonekamp, A. Borisov, G. Borissov, J. Bortfeldt, D. Bortoletto, V. Bortolotto, D. Boscherini, M. Bosman, J. D. Bossio Sola, J. Boudreau, E. V. Bouhova-Thacker, D. Boumediene, C. Bourdarios, S. K. Boutle, A. Boveia, J. Boyd, I. R. Boyko, A. J. Bozson, J. Bracinik, A. Brandt, G. Brandt, O. Brandt, F. Braren, U. Bratzler, B. Brau, J. E. Brau, W. D. Breaden Madden, K. Brendlinger, A. J. Brennan, L. Brenner, R. Brenner, S. Bressler, D. L. Briglin, T. M. Bristow, D. Britton, D. Britzger, F. M. Brochu, I. Brock, R. Brock, G. Brooijmans, T. Brooks, W. K. Brooks, E. Brost, J. H Broughton, P. A. Bruckman de Renstrom, D. Bruncko, A. Bruni, G. Bruni, L. S. Bruni, S. Bruno, BH Brunt, M. Bruschi, N. Bruscino, P. Bryant, L. Bryngemark, T. Buanes, Q. Buat, P. Buchholz, A. G. Buckley, I. A. Budagov, F. Buehrer, M. K. Bugge, O. Bulekov, D. Bullock, T. J. Burch, S. Burdin, C. D. Burgard, A. M. Burger, B. Burghgrave, K. Burka, S. Burke, I. Burmeister, J. T. P. Burr, D. Büscher, V. Büscher, E. Buschmann, P. Bussey, J. M. Butler, C. M. Buttar, J. M. Butterworth, P. Butti, W. Buttinger, A. Buzatu, A. R. Buzykaev, C.-Q. Li, S. Cabrera Urbán, D. Caforio, H. Cai, V. M. Cairo, O. Cakir, N. Calace, P. Calafiura, A. Calandri, G. Calderini, P. Calfayan, G. Callea, L. P. Caloba, S. Calvente Lopez, D. Calvet, S. Calvet, T. P. Calvet, R. Camacho Toro, S. Camarda, P. Camarri, D. Cameron, R. Caminal Armadans, C. Camincher, S. Campana, M. Campanelli, A. Camplani, A. Campoverde, V. Canale, M. Cano Bret, J. Cantero, T. Cao, M. D. M. CapeansGarrido, I. Caprini, M. Caprini, M. Capua, R. M. Carbone, R. Cardarelli, F. Cardillo, I. Carli, T. Carli, G. Carlino, B. T. Carlson, L. Carminati, R. M. D. Carney, S. Caron, E. Carquin, S. Carrá, G. D. Carrillo-Montoya, D. Casadei, M. P. Casado, A. F. Casha, M. Casolino, D. W. Casper, R. Castelijn, V. CastilloGimenez, N. F. Castro, A. Catinaccio, J. R. Catmore, A. Cattai, J. Caudron, V. Cavaliere, E. Cavallaro, D. Cavalli, M. Cavalli-Sforza, V. Cavasinni, E. Celebi, F. Ceradini, L. Cerda Alberich, A. S. Cerqueira, A. Cerri, L. Cerrito, F. Cerutti, A. Cervelli, S. A. Cetin, A. Chafaq, D. Chakraborty, S. K. Chan, W. S. Chan, Y. L. Chan, P. Chang, J. D. Chapman, D. G. Charlton, C. C. Chau, C. A. Chavez Barajas, S. Che, S. Cheatham, A. Chegwidden, S. Chekanov, S. V. Chekulaev, G. A. Chelkov, M. A. Chelstowska, C. Chen, C. Chen, H. Chen, J. Chen, S. Chen, S. Chen, X. Chen, Y. Chen, H. C. Cheng, H. J. Cheng, A. Cheplakov, E. Cheremushkina, R. Cherkaoui El Moursli, E. Cheu, K. Cheung, L. Chevalier, V. Chiarella, G. Chiarelli, G. Chiodini, A. S. Chisholm, A. Chitan, Y. H. Chiu, M. V. Chizhov, K. Choi, A. R. Chomont, S. Chouridou, Y. S. Chow, V. Christodoulou, M. C. Chu, J. Chudoba, A. J. Chuinard, J. J. Chwastowski, L. Chytka, A. K. Ciftci, D. Cinca, V. Cindro, I. A. Cioara, A. Ciocio, F. Cirotto, Z. H. Citron, M. Citterio, M. Ciubancan, A. Clark, M. R. Clark, P. J. Clark, R. N. Clarke, C. Clement, Y. Coadou, M. Cobal, A. Coccaro, J. Cochran, L. Colasurdo, B. Cole, A. P. Colijn, J. Collot, T. Colombo, P. Conde Muiño, E. Coniavitis, S. H. Connell, I. A. Connelly, S. Constantinescu, G. Conti, F. Conventi, A. M. Cooper-Sarkar, F. Cormier, K. J. R. Cormier, M. Corradi, E. E. Corrigan, F. Corriveau, A. Cortes-Gonzalez, G. Costa, M. J. Costa, D. Costanzo, G. Cottin, G. Cowan, B. E. Cox, K. Cranmer, S. J. Crawley, R. A. Creager, G. Cree, S. Crépé-Renaudin, F. Crescioli, W. A. Cribbs, M. Cristinziani, V. Croft, G. Crosetti, A. Cueto, T. CuhadarDonszelmann, A. R. Cukierman, J. Cummings, M. Curatolo, J. Cúth, S. Czekierda, P. Czodrowski, G. D’amen, S. D’Auria, L. D’eramo, M. D’Onofrio, M. J. Da Cunha Sargedas De Sousa, C. Da Via, W. Dabrowski, T. Dado, T. Dai, O. Dale, F. Dallaire, C. Dallapiccola, M. Dam, J. R. Dandoy, M. F. Daneri, N. P. Dang, N. S. Dann, M. Danninger, M. DanoHoffmann, V. Dao, G. Darbo, S. Darmora, J. Dassoulas, A. Dattagupta, T. Daubney, W. Davey, C. David, T. Davidek, D. R. Davis, P. Davison, E. Dawe, I. Dawson, K. De, R. de Asmundis, A. De Benedetti, S. De Castro, S. De Cecco, N. De Groot, P. de Jong, H. De la Torre, F. De Lorenzi, A. De Maria, D. De Pedis, A. De Salvo, U. De Sanctis, A. De Santo, K. De Vasconcelos Corga, J. B. De Vivie De Regie, R. Debbe, C. Debenedetti, D. V. Dedovich, N. Dehghanian, I. Deigaard, M. Del Gaudio, J. Del Peso, D. Delgove, F. Deliot, C. M. Delitzsch, A. Dell’Acqua, L. Dell’Asta, M. Della Pietra, D. della Volpe, M. Delmastro, C. Delporte, P. A. Delsart, D. A. DeMarco, S. Demers, M. Demichev, A. Demilly, S. P. Denisov, D. Denysiuk, D. Derendarz, J. E. Derkaoui, F. Derue, P. Dervan, K. Desch, C. Deterre, K. Dette, M. R. Devesa, P. O. Deviveiros, A. Dewhurst, S. Dhaliwal, F. A. Di Bello, A. Di Ciaccio, L. Di Ciaccio, W. K. Di Clemente, C. Di Donato, A. Di Girolamo, B. Di Girolamo, B. Di Micco, R. Di Nardo, K. F. Di Petrillo, A. Di Simone, R. Di Sipio, D. DiValentino, C. Diaconu, M. Diamond, F. A. Dias, M. A. Diaz, J. Dickinson, E. B. Diehl, J. Dietrich, S. Díez Cornell, A. Dimitrievska, J. Dingfelder, P. Dita, S. Dita, F. Dittus, F. Djama, T. Djobava, J. I. Djuvsland, M. A. B. do Vale, M. Dobre, D. Dodsworth, C. Doglioni, J. Dolejsi, Z. Dolezal, M. Donadelli, S. Donati, J. Donini, J. Dopke, A. Doria, M. T. Dova, A. T. Doyle, E. Drechsler, M. Dris, Y. Du, J. Duarte-Campderros, F. Dubinin, A. Dubreuil, E. Duchovni, G. Duckeck, A. Ducourthial, O. A. Ducu, D. Duda, A. Dudarev, A. Chr. Dudder, E. M. Duffield, L. Duflot, M. Dührssen, C. Dulsen, M. Dumancic, A. E. Dumitriu, A. K. Duncan, M. Dunford, A. Duperrin, H. DuranYildiz, M. Düren, A. Durglishvili, D. Duschinger, B. Dutta, D. Duvnjak, M. Dyndal, B. S. Dziedzic, C. Eckardt, K. M. Ecker, R. C. Edgar, T. Eifert, G. Eigen, K. Einsweiler, T. Ekelof, M. El Kacimi, R. El Kosseifi, V. Ellajosyula, M. Ellert, S. Elles, F. Ellinghaus, A. A. Elliot, N. Ellis, J. Elmsheuser, M. Elsing, D. Emeliyanov, Y. Enari, J. S. Ennis, M. B. Epland, J. Erdmann, A. Ereditato, M. Ernst, S. Errede, M. Escalier, C. Escobar, B. Esposito, O. Estrada Pastor, A. I. Etienvre, E. Etzion, H. Evans, A. Ezhilov, M. Ezzi, F. Fabbri, L. Fabbri, V. Fabiani, G. Facini, R. M. Fakhrutdinov, S. Falciano, R. J. Falla, J. Faltova, Y. Fang, M. Fanti, A. Farbin, A. Farilla, E. M. Farina, T. Farooque, S. Farrell, S. M. Farrington, P. Farthouat, F. Fassi, P. Fassnacht, D. Fassouliotis, M. FaucciGiannelli, A. Favareto, W. J. Fawcett, L. Fayard, O. L. Fedin, W. Fedorko, S. Feigl, L. Feligioni, C. Feng, E. J. Feng, M. Feng, M. J. Fenton, A. B. Fenyuk, L. Feremenga, P. Fernandez Martinez, J. Ferrando, A. Ferrari, P. Ferrari, R. Ferrari, D. E. Ferreira de Lima, A. Ferrer, D. Ferrere, C. Ferretti, F. Fiedler, A. Filipčič, M. Filipuzzi, F. Filthaut, M. Fincke-Keeler, K. D. Finelli, M. C. N. Fiolhais, L. Fiorini, C. Fischer, J. Fischer, W. C. Fisher, N. Flaschel, I. Fleck, P. Fleischmann, R. R. M. Fletcher, T. Flick, B. M. Flierl, L. R. FloresCastillo, G. T. Forcolin, A. Formica, F. A. Förster, A. Forti, A. G. Foster, D. Fournier, H. Fox, S. Fracchia, P. Francavilla, M. Franchini, S. Franchino, D. Francis, L. Franconi, M. Franklin, M. Frate, M. Fraternali, D. Freeborn, S. M. Fressard-Batraneanu, B. Freund, D. Froidevaux, J. A. Frost, C. Fukunaga, T. Fusayasu, J. Fuster, O. Gabizon, A. Gabrielli, A. Gabrielli, G. P. Gach, S. Gadatsch, S. Gadomski, G. Gagliardi, L. G. Gagnon, C. Galea, B. Galhardo, E. J. Gallas, B. J. Gallop, P. Gallus, G. Galster, K. K. Gan, S. Ganguly, Y. Gao, Y. S. Gao, F. M. Garay Walls, C. García, J. E. García Navarro, J. A. García Pascual, M. Garcia-Sciveres, R. W. Gardner, N. Garelli, V. Garonne, A. Gascon Bravo, K. Gasnikova, C. Gatti, A. Gaudiello, G. Gaudio, I. L. Gavrilenko, C. Gay, G. Gaycken, E. N. Gazis, C. N. P. Gee, J. Geisen, M. Geisen, M. P. Geisler, K. Gellerstedt, C. Gemme, M. H. Genest, C. Geng, S. Gentile, C. Gentsos, S. George, D. Gerbaudo, G. Geßner, S. Ghasemi, M. Ghneimat, B. Giacobbe, S. Giagu, N. Giangiacomi, P. Giannetti, S. M. Gibson, M. Gignac, M. Gilchriese, D. Gillberg, G. Gilles, D. M. Gingrich, M. P. Giordani, F. M. Giorgi, P. F. Giraud, P. Giromini, G. Giugliarelli, D. Giugni, F. Giuli, M. Giulini, B. K. Gjelsten, S. Gkaitatzis, I. Gkialas, E. L. Gkougkousis, P. Gkountoumis, L. K. Gladilin, C. Glasman, J. Glatzer, P. C. F. Glaysher, A. Glazov, M. Goblirsch-Kolb, J. Godlewski, S. Goldfarb, T. Golling, D. Golubkov, A. Gomes, R. Gonçalo, R. Goncalves Gama, J. Goncalves Pinto Firmino Da Costa, G. Gonella, L. Gonella, A. Gongadze, F. Gonnella, J. L. Gonski, S. González de laHoz, S. Gonzalez-Sevilla, L. Goossens, P. A. Gorbounov, H. A. Gordon, B. Gorini, E. Gorini, A. Gorišek, A. T. Goshaw, C. Gössling, M. I. Gostkin, C. A. Gottardo, C. R. Goudet, D. Goujdami, A. G. Goussiou, N. Govender, E. Gozani, I. Grabowska-Bold, P. O. J. Gradin, E. C. Graham, J. Gramling, E. Gramstad, S. Grancagnolo, V. Gratchev, P. M. Gravila, C. Gray, H. M. Gray, Z. D. Greenwood, C. Grefe, K. Gregersen, I. M. Gregor, P. Grenier, K. Grevtsov, J. Griffiths, A. A. Grillo, K. Grimm, S. Grinstein, Ph. Gris, J.-F. Grivaz, S. Groh, E. Gross, J. Grosse-Knetter, G. C. Grossi, Z. J. Grout, A. Grummer, L. Guan, W. Guan, J. Guenther, F. Guescini, D. Guest, O. Gueta, B. Gui, E. Guido, T. Guillemin, S. Guindon, U. Gul, C. Gumpert, J. Guo, W. Guo, Y. Guo, R. Gupta, S. Gurbuz, G. Gustavino, B. J. Gutelman, P. Gutierrez, N. G. Gutierrez Ortiz, C. Gutschow, C. Guyot, M. P. Guzik, C. Gwenlan, C. B. Gwilliam, A. Haas, C. Haber, H. K. Hadavand, N. Haddad, A. Hadef, S. Hageböck, M. Hagihara, H. Hakobyan, M. Haleem, J. Haley, G. Halladjian, G. D. Hallewell, K. Hamacher, P. Hamal, K. Hamano, A. Hamilton, G. N. Hamity, P. G. Hamnett, K. Han, L. Han, S. Han, K. Hanagaki, K. Hanawa, M. Hance, D. M. Handl, B. Haney, P. Hanke, J. B. Hansen, J. D. Hansen, M. C. Hansen, P. H. Hansen, K. Hara, A. S. Hard, T. Harenberg, F. Hariri, S. Harkusha, P. F. Harrison, N. M. Hartmann, Y. Hasegawa, A. Hasib, S. Hassani, S. Haug, R. Hauser, L. Hauswald, L. B. Havener, M. Havranek, C. M. Hawkes, R. J. Hawkings, D. Hayden, C. P. Hays, J. M. Hays, H. S. Hayward, S. J. Haywood, T. Heck, V. Hedberg, L. Heelan, S. Heer, K. K. Heidegger, S. Heim, T. Heim, B. Heinemann, J. J. Heinrich, L. Heinrich, C. Heinz, J. Hejbal, L. Helary, A. Held, S. Hellman, C. Helsens, R. C. W. Henderson, Y. Heng, S. Henkelmann, A. M. Henriques Correia, S. Henrot-Versille, G. H. Herbert, H. Herde, V. Herget, Y. Hernández Jiménez, H. Herr, G. Herten, R. Hertenberger, L. Hervas, T. C. Herwig, G. G. Hesketh, N. P. Hessey, J. W. Hetherly, S. Higashino, E. Higón-Rodriguez, K. Hildebrand, E. Hill, J. C. Hill, K. H. Hiller, S. J. Hillier, M. Hils, I. Hinchliffe, M. Hirose, D. Hirschbuehl, B. Hiti, O. Hladik, D. R. Hlaluku, X. Hoad, J. Hobbs, N. Hod, M. C. Hodgkinson, P. Hodgson, A. Hoecker, M. R. Hoeferkamp, F. Hoenig, D. Hohn, T. R. Holmes, M. Holzbock, M. Homann, S. Honda, T. Honda, T. M. Hong, B. H. Hooberman, W. H. Hopkins, Y. Horii, A. J. Horton, J-Y. Hostachy, A. Hostiuc, S. Hou, A. Hoummada, J. Howarth, J. Hoya, M. Hrabovsky, J. Hrdinka, I. Hristova, J. Hrivnac, T. Hryn’ova, A. Hrynevich, P. J. Hsu, S.-C. Hsu, Q. Hu, S. Hu, Y. Huang, Z. Hubacek, F. Hubaut, F. Huegging, T. B. Huffman, E. W. Hughes, M. Huhtinen, R. F. H. Hunter, P. Huo, N. Huseynov, J. Huston, J. Huth, R. Hyneman, G. Iacobucci, G. Iakovidis, I. Ibragimov, L. Iconomidou-Fayard, Z. Idrissi, P. Iengo, O. Igonkina, T. Iizawa, Y. Ikegami, M. Ikeno, Y. Ilchenko, D. Iliadis, N. Ilic, F. Iltzsche, G. Introzzi, P. Ioannou, M. Iodice, K. Iordanidou, V. Ippolito, M. F. Isacson, N. Ishijima, M. Ishino, M. Ishitsuka, C. Issever, S. Istin, F. Ito, J. M. Iturbe Ponce, R. Iuppa, H. Iwasaki, J. M. Izen, V. Izzo, S. Jabbar, P. Jackson, R. M. Jacobs, V. Jain, K. B. Jakobi, K. Jakobs, S. Jakobsen, T. Jakoubek, D. O. Jamin, D. K. Jana, R. Jansky, J. Janssen, M. Janus, P. A. Janus, G. Jarlskog, N. Javadov, T. Javůrek, M. Javurkova, F. Jeanneau, L. Jeanty, J. Jejelava, A. Jelinskas, P. Jenni, C. Jeske, S. Jézéquel, H. Ji, J. Jia, H. Jiang, Y. Jiang, Z. Jiang, S. Jiggins, J. Jimenez Pena, S. Jin, A. Jinaru, O. Jinnouchi, H. Jivan, P. Johansson, K. A. Johns, C. A. Johnson, W. J. Johnson, K. Jon-And, R. W. L. Jones, S. D. Jones, S. Jones, T. J. Jones, J. Jongmanns, P. M. Jorge, J. Jovicevic, X. Ju, A. Juste Rozas, M. K. Köhler, A. Kaczmarska, M. Kado, H. Kagan, M. Kagan, S. J. Kahn, T. Kaji, E. Kajomovitz, C. W. Kalderon, A. Kaluza, S. Kama, A. Kamenshchikov, N. Kanaya, L. Kanjir, V. A. Kantserov, J. Kanzaki, B. Kaplan, L. S. Kaplan, D. Kar, K. Karakostas, N. Karastathis, M. J. Kareem, E. Karentzos, S. N. Karpov, Z. M. Karpova, V. Kartvelishvili, A. N. Karyukhin, K. Kasahara, L. Kashif, R. D. Kass, A. Kastanas, Y. Kataoka, C. Kato, A. Katre, J. Katzy, K. Kawade, K. Kawagoe, T. Kawamoto, G. Kawamura, E. F. Kay, V. F. Kazanin, R. Keeler, R. Kehoe, J. S. Keller, E. Kellermann, J. J. Kempster, J Kendrick, H. Keoshkerian, O. Kepka, B. P. Kerševan, S. Kersten, R. A. Keyes, M. Khader, F. Khalil-zada, A. Khanov, A. G. Kharlamov, T. Kharlamova, A. Khodinov, T. J. Khoo, V. Khovanskiy, E. Khramov, J. Khubua, S. Kido, M. Kiehn, C. R. Kilby, H. Y. Kim, S. H. Kim, Y. K. Kim, N. Kimura, O. M. Kind, B. T. King, D. Kirchmeier, J. Kirk, A. E. Kiryunin, T. Kishimoto, D. Kisielewska, V. Kitali, O. Kivernyk, E. Kladiva, T. Klapdor-Kleingrothaus, M. H. Klein, M. Klein, U. Klein, K. Kleinknecht, P. Klimek, A. Klimentov, R. Klingenberg, T. Klingl, T. Klioutchnikova, F. F. Klitzner, E.-E. Kluge, P. Kluit, S. Kluth, E. Kneringer, E. B. F. G. Knoops, A. Knue, A. Kobayashi, D. Kobayashi, T. Kobayashi, M. Kobel, M. Kocian, P. Kodys, T. Koffas, E. Koffeman, N. M. Köhler, T. Koi, M. Kolb, I. Koletsou, T. Kondo, N. Kondrashova, K. Köneke, A. C. König, T. Kono, R. Konoplich, N. Konstantinidis, B. Konya, R. Kopeliansky, S. Koperny, K. Korcyl, K. Kordas, A. Korn, I. Korolkov, E. V. Korolkova, O. Kortner, S. Kortner, T. Kosek, V. V. Kostyukhin, A. Kotwal, A. Koulouris, A. Kourkoumeli-Charalampidi, C. Kourkoumelis, E. Kourlitis, V. Kouskoura, A. B. Kowalewska, R. Kowalewski, T. Z. Kowalski, C. Kozakai, W. Kozanecki, A. S. Kozhin, V. A. Kramarenko, G. Kramberger, D. Krasnopevtsev, M. W. Krasny, A. Krasznahorkay, D. Krauss, J. A. Kremer, J. Kretzschmar, K. Kreutzfeldt, P. Krieger, K. Krizka, K. Kroeninger, H. Kroha, J. Kroll, J. Kroll, J. Kroseberg, J. Krstic, U. Kruchonak, H. Krüger, N. Krumnack, M. C. Kruse, T. Kubota, H. Kucuk, S. Kuday, J. T. Kuechler, S. Kuehn, A. Kugel, F. Kuger, T. Kuhl, V. Kukhtin, R. Kukla, Y. Kulchitsky, S. Kuleshov, Y. P. Kulinich, M. Kuna, T. Kunigo, A. Kupco, T. Kupfer, O. Kuprash, H. Kurashige, L. L. Kurchaninov, Y. A. Kurochkin, M. G. Kurth, E. S. Kuwertz, M. Kuze, J. Kvita, T. Kwan, D. Kyriazopoulos, A. La Rosa, J. L. La Rosa Navarro, L. La Rotonda, F. La Ruffa, C. Lacasta, F. Lacava, J. Lacey, D. P. J. Lack, H. Lacker, D. Lacour, E. Ladygin, R. Lafaye, B. Laforge, S. Lai, S. Lammers, W. Lampl, E. Lançon, U. Landgraf, M. P. J. Landon, M. C. Lanfermann, V. S. Lang, J. C. Lange, R. J. Langenberg, A. J. Lankford, F. Lanni, K. Lantzsch, A. Lanza, A. Lapertosa, S. Laplace, J. F. Laporte, T. Lari, F. Lasagni Manghi, M. Lassnig, T. S. Lau, P. Laurelli, W. Lavrijsen, A. T. Law, P. Laycock, T. Lazovich, M. Lazzaroni, B. Le, O. Le Dortz, E. Le Guirriec, E. P. Le Quilleuc, M. LeBlanc, T. LeCompte, F. Ledroit-Guillon, C. A. Lee, G. R. Lee, S. C. Lee, L. Lee, B. Lefebvre, G. Lefebvre, M. Lefebvre, F. Legger, C. Leggett, G. Lehmann Miotto, X. Lei, W. A. Leight, M. A. L. Leite, R. Leitner, D. Lellouch, B. Lemmer, K. J. C. Leney, T. Lenz, B. Lenzi, R. Leone, S. Leone, C. Leonidopoulos, G. Lerner, C. Leroy, R. Les, A. A. J. Lesage, C. G. Lester, M. Levchenko, J. Levêque, D. Levin, L. J. Levinson, M. Levy, D. Lewis, B. Li, H. Li, L. Li, Q. Li, Q. Li, S. Li, X. Li, Y. Li, Z. Liang, B. Liberti, A. Liblong, K. Lie, A. Limosani, C. Y. Lin, K. Lin, S. C. Lin, T. H. Lin, R. A. Linck, B. E. Lindquist, A. E. Lionti, E. Lipeles, A. Lipniacka, M. Lisovyi, T. M. Liss, A. Lister, A. M. Litke, B. Liu, H. Liu, H. Liu, J. K. K. Liu, J. Liu, J. B. Liu, K. Liu, L. Liu, M. Liu, Y. L. Liu, Y. Liu, M. Livan, A. Lleres, J. LlorenteMerino, S. L. Lloyd, C. Y. Lo, F. Lo Sterzo, E. M. Lobodzinska, P. Loch, F. K. Loebinger, A. Loesle, K. M. Loew, T. Lohse, K. Lohwasser, M. Lokajicek, B. A. Long, J. D. Long, R. E. Long, L. Longo, K. A. Looper, J. A. Lopez, I. Lopez Paz, A. Lopez Solis, J. Lorenz, N. Lorenzo Martinez, M. Losada, P. J. Lösel, X. Lou, A. Lounis, J. Love, P. A. Love, H. Lu, N. Lu, Y. J. Lu, H. J. Lubatti, C. Luci, A. Lucotte, C. Luedtke, F. Luehring, W. Lukas, L. Luminari, B. Lund-Jensen, M. S. Lutz, P. M. Luzi, D. Lynn, R. Lysak, E. Lytken, F. Lyu, V. Lyubushkin, H. Ma, L. L. Ma, Y. Ma, G. Maccarrone, A. Macchiolo, C. M. Macdonald, B. Maček, J. Machado Miguens, D. Madaffari, R. Madar, W. F. Mader, A. Madsen, N. Madysa, J. Maeda, S. Maeland, T. Maeno, A. S. Maevskiy, V. Magerl, C. Maiani, C. Maidantchik, T. Maier, A. Maio, O. Majersky, S. Majewski, Y. Makida, N. Makovec, B. Malaescu, Pa. Malecki, V. P. Maleev, F. Malek, U. Mallik, D. Malon, C. Malone, S. Maltezos, S. Malyukov, J. Mamuzic, G. Mancini, I. Mandić, J. Maneira, L. Manhaes de Andrade Filho, J. Manjarres Ramos, K. H. Mankinen, A. Mann, A. Manousos, B. Mansoulie, J. D. Mansour, R. Mantifel, M. Mantoani, S. Manzoni, L. Mapelli, G. Marceca, L. March, L. Marchese, G. Marchiori, M. Marcisovsky, C. A. Marin Tobon, M. Marjanovic, D. E. Marley, F. Marroquim, S. P. Marsden, Z. Marshall, M. U. F Martensson, S. Marti-Garcia, C. B. Martin, T. A. Martin, V. J. Martin, B. Martin dit Latour, M. Martinez, V. I. Martinez Outschoorn, S. Martin-Haugh, V. S. Martoiu, A. C. Martyniuk, A. Marzin, L. Masetti, T. Mashimo, R. Mashinistov, J. Masik, A. L. Maslennikov, L. H. Mason, L. Massa, P. Mastrandrea, A. Mastroberardino, T. Masubuchi, P. Mättig, J. Maurer, S. J. Maxfield, D. A. Maximov, R. Mazini, I. Maznas, S. M. Mazza, N. C. Mc Fadden, G. Mc Goldrick, S. P. Mc Kee, A. McCarn, R. L. McCarthy, T. G. McCarthy, L. I. McClymont, E. F. McDonald, J. A. Mcfayden, G. Mchedlidze, S. J. McMahon, P. C. McNamara, C. J. McNicol, R. A. McPherson, S. Meehan, T. J. Megy, S. Mehlhase, A. Mehta, T. Meideck, K. Meier, B. Meirose, D. Melini, B. R. MelladoGarcia, J. D. Mellenthin, M. Melo, F. Meloni, A. Melzer, S. B. Menary, L. Meng, X. T. Meng, A. Mengarelli, S. Menke, E. Meoni, S. Mergelmeyer, C. Merlassino, P. Mermod, L. Merola, C. Meroni, F. S. Merritt, A. Messina, J. Metcalfe, A. S. Mete, C. Meyer, J-P. Meyer, J. Meyer, H. Meyer Zu Theenhausen, F. Miano, R. P. Middleton, S. Miglioranzi, L. Mijović, G. Mikenberg, M. Mikestikova, M. Mikuž, M. Milesi, A. Milic, D. A. Millar, D. W. Miller, C. Mills, A. Milov, D. A. Milstead, A. A. Minaenko, Y. Minami, I. A. Minashvili, A. I. Mincer, B. Mindur, M. Mineev, Y. Minegishi, Y. Ming, L. M. Mir, A. Mirto, K. P. Mistry, T. Mitani, J. Mitrevski, V. A. Mitsou, A. Miucci, P. S. Miyagawa, A. Mizukami, J. U. Mjörnmark, T. Mkrtchyan, M. Mlynarikova, T. Moa, K. Mochizuki, P. Mogg, S. Mohapatra, S. Molander, R. Moles-Valls, M. C. Mondragon, K. Mönig, J. Monk, E. Monnier, A. Montalbano, J. Montejo Berlingen, F. Monticelli, S. Monzani, R. W. Moore, N. Morange, D. Moreno, M. Moreno Llácer, P. Morettini, M. Morgenstern, S. Morgenstern, D. Mori, T. Mori, M. Morii, M. Morinaga, V. Morisbak, A. K. Morley, G. Mornacchi, J. D. Morris, L. Morvaj, P. Moschovakos, M. Mosidze, H. J. Moss, J. Moss, K. Motohashi, R. Mount, E. Mountricha, E. J. W. Moyse, S. Muanza, F. Mueller, J. Mueller, R. S. P. Mueller, D. Muenstermann, P. Mullen, G. A. Mullier, F. J. Munoz Sanchez, W. J. Murray, H. Musheghyan, M. Muškinja, C. Mwewa, A. G. Myagkov, M. Myska, B. P. Nachman, O. Nackenhorst, K. Nagai, R. Nagai, K. Nagano, Y. Nagasaka, K. Nagata, M. Nagel, E. Nagy, A. M. Nairz, Y. Nakahama, K. Nakamura, T. Nakamura, I. Nakano, R. F. Naranjo Garcia, R. Narayan, D. I. Narrias Villar, I. Naryshkin, T. Naumann, G. Navarro, R. Nayyar, H. A. Neal, P. Yu. Nechaeva, T. J. Neep, A. Negri, M. Negrini, S. Nektarijevic, C. Nellist, A. Nelson, M. E. Nelson, S. Nemecek, P. Nemethy, M. Nessi, M. S. Neubauer, M. Neumann, P. R. Newman, T. Y. Ng, Y. S. Ng, T. Nguyen Manh, R. B. Nickerson, R. Nicolaidou, J. Nielsen, N. Nikiforou, V. Nikolaenko, I. Nikolic-Audit, K. Nikolopoulos, P. Nilsson, Y. Ninomiya, A. Nisati, N. Nishu, R. Nisius, I. Nitsche, T. Nitta, T. Nobe, Y. Noguchi, M. Nomachi, I. Nomidis, M. A. Nomura, T. Nooney, M. Nordberg, N. Norjoharuddeen, O. Novgorodova, M. Nozaki, L. Nozka, K. Ntekas, E. Nurse, F. Nuti, K. O’connor, D. C. O’Neil, A. A. O’Rourke, V. O’Shea, F. G. Oakham, H. Oberlack, T. Obermann, J. Ocariz, A. Ochi, I. Ochoa, J. P. Ochoa-Ricoux, S. Oda, S. Odaka, A. Oh, S. H. Oh, C. C. Ohm, H. Ohman, H. Oide, H. Okawa, Y. Okumura, T. Okuyama, A. Olariu, L. F. Oleiro Seabra, S. A. Olivares Pino, D. Oliveira Damazio, M. J. R. Olsson, A. Olszewski, J. Olszowska, A. Onofre, K. Onogi, P. U. E. Onyisi, H. Oppen, M. J. Oreglia, Y. Oren, D. Orestano, E. C. Orgill, N. Orlando, R. S. Orr, B. Osculati, R. Ospanov, G. Otero y Garzon, H. Otono, M. Ouchrif, F. Ould-Saada, A. Ouraou, K. P. Oussoren, Q. Ouyang, M. Owen, R. E. Owen, V. E. Ozcan, N. Ozturk, K. Pachal, A. Pacheco Pages, L. Pacheco Rodriguez, C. Padilla Aranda, S. Pagan Griso, M. Paganini, F. Paige, G. Palacino, S. Palazzo, S. Palestini, M. Palka, D. Pallin, E. St. Panagiotopoulou, I. Panagoulias, C. E. Pandini, J. G. Panduro Vazquez, P. Pani, S. Panitkin, D. Pantea, L. Paolozzi, Th. D. Papadopoulou, K. Papageorgiou, A. Paramonov, D. Paredes Hernandez, A. J. Parker, M. A. Parker, K. A. Parker, F. Parodi, J. A. Parsons, U. Parzefall, V. R. Pascuzzi, J. M. Pasner, E. Pasqualucci, S. Passaggio, Fr. Pastore, S. Pataraia, J. R. Pater, T. Pauly, B. Pearson, S. Pedraza Lopez, R. Pedro, S. V. Peleganchuk, O. Penc, C. Peng, H. Peng, J. Penwell, B. S. Peralva, M. M. Perego, D. V. Perepelitsa, F. Peri, L. Perini, H. Pernegger, S. Perrella, R. Peschke, V. D. Peshekhonov, K. Peters, R. F. Y. Peters, B. A. Petersen, T. C. Petersen, E. Petit, A. Petridis, C. Petridou, P. Petroff, E. Petrolo, M. Petrov, F. Petrucci, N. E. Pettersson, A. Peyaud, R. Pezoa, F. H. Phillips, P. W. Phillips, G. Piacquadio, E. Pianori, A. Picazio, M. A. Pickering, R. Piegaia, J. E. Pilcher, A. D. Pilkington, M. Pinamonti, J. L. Pinfold, H. Pirumov, M. Pitt, L. Plazak, M.-A. Pleier, V. Pleskot, E. Plotnikova, D. Pluth, P. Podberezko, R. Poettgen, R. Poggi, L. Poggioli, I. Pogrebnyak, D. Pohl, I. Pokharel, G. Polesello, A. Poley, A. Policicchio, R. Polifka, A. Polini, C. S. Pollard, V. Polychronakos, K. Pommès, D. Ponomarenko, L. Pontecorvo, G. A. Popeneciu, D. M. Portillo Quintero, S. Pospisil, K. Potamianos, I. N. Potrap, C. J. Potter, H. Potti, T. Poulsen, J. Poveda, M. E. Pozo Astigarraga, P. Pralavorio, A. Pranko, S. Prell, D. Price, M. Primavera, S. Prince, N. Proklova, K. Prokofiev, F. Prokoshin, S. Protopopescu, J. Proudfoot, M. Przybycien, A. Puri, P. Puzo, J. Qian, Y. Qin, A. Quadt, M. Queitsch-Maitland, D. Quilty, S. Raddum, V. Radeka, V. Radescu, S. K. Radhakrishnan, P. Radloff, P. Rados, F. Ragusa, G. Rahal, J. A. Raine, S. Rajagopalan, T. Rashid, S. Raspopov, M. G. Ratti, D. M. Rauch, F. Rauscher, S. Rave, I. Ravinovich, J. H. Rawling, M. Raymond, A. L. Read, N. P. Readioff, M. Reale, D. M. Rebuzzi, A. Redelbach, G. Redlinger, R. Reece, R. G. Reed, K. Reeves, L. Rehnisch, J. Reichert, A. Reiss, C. Rembser, H. Ren, M. Rescigno, S. Resconi, E. D. Resseguie, S. Rettie, E. Reynolds, O. L. Rezanova, P. Reznicek, R. Rezvani, R. Richter, S. Richter, E. Richter-Was, O. Ricken, M. Ridel, P. Rieck, C. J. Riegel, J. Rieger, O. Rifki, M. Rijssenbeek, A. Rimoldi, M. Rimoldi, L. Rinaldi, G. Ripellino, B. Ristić, E. Ritsch, I. Riu, F. Rizatdinova, E. Rizvi, C. Rizzi, R. T. Roberts, S. H. Robertson, A. Robichaud-Veronneau, D. Robinson, J. E. M. Robinson, A. Robson, E. Rocco, C. Roda, Y. Rodina, S. Rodriguez Bosca, A. Rodriguez Perez, D. Rodriguez Rodriguez, S. Roe, C. S. Rogan, O. Røhne, J. Roloff, A. Romaniouk, M. Romano, S. M. RomanoSaez, E. Romero Adam, N. Rompotis, M. Ronzani, L. Roos, S. Rosati, K. Rosbach, P. Rose, N.-A. Rosien, E. Rossi, L. P. Rossi, J. H. N. Rosten, R. Rosten, M. Rotaru, J. Rothberg, D. Rousseau, D. Roy, A. Rozanov, Y. Rozen, X. Ruan, F. Rubbo, F. Rühr, A. Ruiz-Martinez, Z. Rurikova, N. A. Rusakovich, H. L. Russell, J. P. Rutherfoord, N. Ruthmann, E. M. Rüttinger, Y. F. Ryabov, M. Rybar, G. Rybkin, S. Ryu, A. Ryzhov, G. F. Rzehorz, A. F. Saavedra, G. Sabato, S. Sacerdoti, H.F-W. Sadrozinski, R. Sadykov, F. Safai Tehrani, P. Saha, M. Sahinsoy, M. Saimpert, M. Saito, T. Saito, H. Sakamoto, Y. Sakurai, G. Salamanna, J. E. Salazar Loyola, D. Salek, P. H. Sales De Bruin, D. Salihagic, A. Salnikov, J. Salt, D. Salvatore, F. Salvatore, A. Salvucci, A. Salzburger, D. Sammel, D. Sampsonidis, D. Sampsonidou, J. Sánchez, A. SanchezPineda, H. Sandaker, R. L. Sandbach, C. O. Sander, M. Sandhoff, C. Sandoval, D. P. C. Sankey, M. Sannino, Y. Sano, A. Sansoni, C. Santoni, H. Santos, I. Santoyo Castillo, A. Sapronov, J. G. Saraiva, O. Sasaki, K. Sato, E. Sauvan, G. Savage, P. Savard, N. Savic, C. Sawyer, L. Sawyer, C. Sbarra, A. Sbrizzi, T. Scanlon, D. A. Scannicchio, J. Schaarschmidt, P. Schacht, B. M. Schachtner, D. Schaefer, L. Schaefer, J. Schaeffer, S. Schaepe, U. Schäfer, A. C. Schaffer, D. Schaile, R. D. Schamberger, V. A. Schegelsky, D. Scheirich, F. Schenck, M. Schernau, C. Schiavi, S. Schier, L. K. Schildgen, C. Schillo, M. Schioppa, S. Schlenker, K. R. Schmidt-Sommerfeld, K. Schmieden, C. Schmitt, S. Schmitt, S. Schmitz, U. Schnoor, L. Schoeffel, A. Schoening, B. D. Schoenrock, E. Schopf, M. Schott, J. F. P. Schouwenberg, J. Schovancova, S. Schramm, N. Schuh, A. Schulte, M. J. Schultens, H.-C. Schultz-Coulon, M. Schumacher, B. A. Schumm, Ph. Schune, A. Schwartzman, T. A. Schwarz, H. Schweiger, Ph. Schwemling, R. Schwienhorst, J. Schwindling, A. Sciandra, G. Sciolla, M. Scornajenghi, F. Scuri, F. Scutti, J. Searcy, P. Seema, S. C. Seidel, A. Seiden, J. M. Seixas, G. Sekhniaidze, K. Sekhon, S. J. Sekula, N. Semprini-Cesari, S. Senkin, C. Serfon, L. Serin, L. Serkin, M. Sessa, R. Seuster, H. Severini, T. Šfiligoj, F. Sforza, A. Sfyrla, E. Shabalina, N. W. Shaikh, L. Y. Shan, R. Shang, J. T. Shank, M. Shapiro, P. B. Shatalov, K. Shaw, S. M. Shaw, A. Shcherbakova, C. Y. Shehu, Y. Shen, N. Sherafati, A. D. Sherman, P. Sherwood, L. Shi, S. Shimizu, C. O. Shimmin, M. Shimojima, I. P. J. Shipsey, S. Shirabe, M. Shiyakova, J. Shlomi, A. Shmeleva, D. Shoaleh Saadi, M. J. Shochet, S. Shojaii, D. R. Shope, S. Shrestha, E. Shulga, M. A. Shupe, P. Sicho, A. M. Sickles, P. E. Sidebo, E. Sideras Haddad, O. Sidiropoulou, A. Sidoti, F. Siegert, Dj. Sijacki, J. Silva, S. B. Silverstein, V. Simak, L. Simic, S. Simion, E. Simioni, B. Simmons, M. Simon, P. Sinervo, N. B. Sinev, M. Sioli, G. Siragusa, I. Siral, S. Yu. Sivoklokov, J. Sjölin, M. B. Skinner, P. Skubic, M. Slater, T. Slavicek, M. Slawinska, K. Sliwa, R. Slovak, V. Smakhtin, B. H. Smart, J. Smiesko, N. Smirnov, S. Yu. Smirnov, Y. Smirnov, L. N. Smirnova, O. Smirnova, J. W. Smith, M. N. K. Smith, R. W. Smith, M. Smizanska, K. Smolek, A. A. Snesarev, I. M. Snyder, S. Snyder, R. Sobie, F. Socher, A. Soffer, A. Søgaard, D. A. Soh, G. Sokhrannyi, C. A. Solans Sanchez, M. Solar, E. Yu. Soldatov, U. Soldevila, A. A. Solodkov, A. Soloshenko, O. V. Solovyanov, V. Solovyev, P. Sommer, H. Son, A. Sopczak, D. Sosa, C. L. Sotiropoulou, S. Sottocornola, R. Soualah, A. M. Soukharev, D. South, B. C. Sowden, S. Spagnolo, M. Spalla, M. Spangenberg, F. Spanò, D. Sperlich, F. Spettel, T. M. Spieker, R. Spighi, G. Spigo, L. A. Spiller, M. Spousta, R. D. St.Denis, A. Stabile, R. Stamen, S. Stamm, E. Stanecka, R. W. Stanek, C. Stanescu, M. M. Stanitzki, B. S. Stapf, S. Stapnes, E. A. Starchenko, G. H. Stark, J. Stark, S. H Stark, P. Staroba, P. Starovoitov, S. Stärz, R. Staszewski, M. Stegler, P. Steinberg, B. Stelzer, H. J. Stelzer, O. Stelzer-Chilton, H. Stenzel, T. J. Stevenson, G. A. Stewart, M. C. Stockton, M. Stoebe, G. Stoicea, P. Stolte, S. Stonjek, A. R. Stradling, A. Straessner, M. E. Stramaglia, J. Strandberg, S. Strandberg, M. Strauss, P. Strizenec, R. Ströhmer, D. M. Strom, R. Stroynowski, A. Strubig, S. A. Stucci, B. Stugu, N. A. Styles, D. Su, J. Su, S. Suchek, Y. Sugaya, M. Suk, V. V. Sulin, DMS Sultan, S. Sultansoy, T. Sumida, S. Sun, X. Sun, K. Suruliz, C. J. E. Suster, M. R. Sutton, S. Suzuki, M. Svatos, M. Swiatlowski, S. P. Swift, I. Sykora, T. Sykora, D. Ta, K. Tackmann, J. Taenzer, A. Taffard, R. Tafirout, E. Tahirovic, N. Taiblum, H. Takai, R. Takashima, E. H. Takasugi, K. Takeda, T. Takeshita, Y. Takubo, M. Talby, A. A. Talyshev, J. Tanaka, M. Tanaka, R. Tanaka, R. Tanioka, B. B. Tannenwald, S. Tapia Araya, S. Tapprogge, S. Tarem, G. F. Tartarelli, P. Tas, M. Tasevsky, T. Tashiro, E. Tassi, A. Tavares Delgado, Y. Tayalati, A. C. Taylor, A. J. Taylor, G. N. Taylor, P. T. E. Taylor, W. Taylor, P. Teixeira-Dias, D. Temple, H. Ten Kate, P. K. Teng, J. J. Teoh, F. Tepel, S. Terada, K. Terashi, J. Terron, S. Terzo, M. Testa, R. J. Teuscher, S. J. Thais, T. Theveneaux-Pelzer, F. Thiele, J. P. Thomas, J. Thomas-Wilsker, P. D. Thompson, A. S. Thompson, L. A. Thomsen, E. Thomson, Y. Tian, M. J. Tibbetts, R. E. Ticse Torres, V. O. Tikhomirov, Yu. A. Tikhonov, S. Timoshenko, P. Tipton, S. Tisserant, K. Todome, S. Todorova-Nova, S. Todt, J. Tojo, S. Tokár, K. Tokushuku, E. Tolley, L. Tomlinson, M. Tomoto, L. Tompkins, K. Toms, B. Tong, P. Tornambe, E. Torrence, H. Torres, E. Torró Pastor, J. Toth, F. Touchard, D. R. Tovey, C. J. Treado, T. Trefzger, F. Tresoldi, A. Tricoli, I. M. Trigger, S. Trincaz-Duvoid, M. F. Tripiana, W. Trischuk, B. Trocmé, A. Trofymov, C. Troncon, M. Trovatelli, L. Truong, M. Trzebinski, A. Trzupek, K. W. Tsang, J.C-L. Tseng, P. V. Tsiareshka, N. Tsirintanis, S. Tsiskaridze, V. Tsiskaridze, E. G. Tskhadadze, I. I. Tsukerman, V. Tsulaia, S. Tsuno, D. Tsybychev, Y. Tu, A. Tudorache, V. Tudorache, T. T. Tulbure, A. N. Tuna, S. Turchikhin, D. Turgeman, I. Turk Cakir, R. Turra, P. M. Tuts, G. Ucchielli, I. Ueda, M. Ughetto, F. Ukegawa, G. Unal, A. Undrus, G. Unel, F. C. Ungaro, Y. Unno, K. Uno, J. Urban, P. Urquijo, P. Urrejola, G. Usai, J. Usui, L. Vacavant, V. Vacek, B. Vachon, K. O. H. Vadla, A. Vaidya, C. Valderanis, E. ValdesSanturio, M. Valente, S. Valentinetti, A. Valero, L. Valéry, A. Vallier, J. A. Valls Ferrer, W. Van Den Wollenberg, H. van der Graaf, P. van Gemmeren, J. Van Nieuwkoop, I. van Vulpen, M. C. van Woerden, M. Vanadia, W. Vandelli, A. Vaniachine, P. Vankov, G. Vardanyan, R. Vari, E. W. Varnes, C. Varni, T. Varol, D. Varouchas, A. Vartapetian, K. E. Varvell, J. G. Vasquez, G. A. Vasquez, F. Vazeille, D. Vazquez Furelos, T. Vazquez Schroeder, J. Veatch, V. Veeraraghavan, L. M. Veloce, F. Veloso, S. Veneziano, A. Ventura, M. Venturi, N. Venturi, V. Vercesi, M. Verducci, W. Verkerke, A. T. Vermeulen, J. C. Vermeulen, M. C. Vetterli, N. Viaux Maira, O. Viazlo, I. Vichou, T. Vickey, O. E. Vickey Boeriu, G. H. A. Viehhauser, S. Viel, L. Vigani, M. Villa, M. Villaplana Perez, E. Vilucchi, M. G. Vincter, V. B. Vinogradov, A. Vishwakarma, C. Vittori, I. Vivarelli, S. Vlachos, M. Vogel, P. Vokac, G. Volpi, S. E. von Buddenbrock, H. von der Schmitt, E. von Toerne, V. Vorobel, K. Vorobev, M. Vos, R. Voss, J. H. Vossebeld, N. Vranjes, M. Vranjes Milosavljevic, V. Vrba, M. Vreeswijk, R. Vuillermet, I. Vukotic, P. Wagner, W. Wagner, J. Wagner-Kuhr, H. Wahlberg, S. Wahrmund, K. Wakamiya, J. Walder, R. Walker, W. Walkowiak, V. Wallangen, A. M. Wang, C. Wang, F. Wang, H. Wang, H. Wang, J. Wang, J. Wang, Q. Wang, R.-J. Wang, R. Wang, S. M. Wang, T. Wang, W. Wang, W. Wang, Z. Wang, C. Wanotayaroj, A. Warburton, C. P. Ward, D. R. Wardrope, A. Washbrook, P. M. Watkins, A. T. Watson, M. F. Watson, G. Watts, S. Watts, B. M. Waugh, A. F. Webb, S. Webb, M. S. Weber, S. M. Weber, S. A. Weber, J. S. Webster, A. R. Weidberg, B. Weinert, J. Weingarten, M. Weirich, C. Weiser, P. S. Wells, T. Wenaus, T. Wengler, S. Wenig, N. Wermes, M. D. Werner, P. Werner, M. Wessels, T. D. Weston, K. Whalen, N. L. Whallon, A. M. Wharton, A. S. White, A. White, M. J. White, R. White, D. Whiteson, B. W. Whitmore, F. J. Wickens, W. Wiedenmann, M. Wielers, C. Wiglesworth, L. A. M. Wiik-Fuchs, A. Wildauer, F. Wilk, H. G. Wilkens, H. H. Williams, S. Williams, C. Willis, S. Willocq, J. A. Wilson, I. Wingerter-Seez, E. Winkels, F. Winklmeier, O. J. Winston, B. T. Winter, M. Wittgen, M. Wobisch, A. Wolf, T. M. H. Wolf, R. Wolff, M. W. Wolter, H. Wolters, V. W. S. Wong, N. L. Woods, S. D. Worm, B. K. Wosiek, J. Wotschack, K. W. Wozniak, M. Wu, S. L. Wu, X. Wu, Y. Wu, T. R. Wyatt, B. M. Wynne, S. Xella, Z. Xi, L. Xia, D. Xu, L. Xu, T. Xu, W. Xu, B. Yabsley, S. Yacoob, K. Yajima, D. Yamaguchi, Y. Yamaguchi, A. Yamamoto, S. Yamamoto, T. Yamanaka, F. Yamane, M. Yamatani, T. Yamazaki, Y. Yamazaki, Z. Yan, H. Yang, H. Yang, Y. Yang, Z. Yang, W-M. Yao, Y. C. Yap, Y. Yasu, E. Yatsenko, K. H. YauWong, J. Ye, S. Ye, I. Yeletskikh, E. Yigitbasi, E. Yildirim, K. Yorita, K. Yoshihara, C. Young, C. J. S. Young, J. Yu, J. Yu, S. P. Y. Yuen, I. Yusuff, B. Zabinski, G. Zacharis, R. Zaidan, A. M. Zaitsev, N. Zakharchuk, J. Zalieckas, A. Zaman, S. Zambito, D. Zanzi, C. Zeitnitz, G. Zemaityte, J. C. Zeng, Q. Zeng, O. Zenin, T. Ženiš, D. Zerwas, D. Zhang, D. Zhang, F. Zhang, G. Zhang, H. Zhang, J. Zhang, L. Zhang, L. Zhang, M. Zhang, P. Zhang, R. Zhang, R. Zhang, X. Zhang, Y. Zhang, Z. Zhang, X. Zhao, Y. Zhao, Z. Zhao, A. Zhemchugov, B. Zhou, C. Zhou, L. Zhou, M. Zhou, M. Zhou, N. Zhou, Y. Zhou, C. G. Zhu, H. Zhu, J. Zhu, Y. Zhu, X. Zhuang, K. Zhukov, A. Zibell, D. Zieminska, N. I. Zimine, S. Zimmermann, Z. Zinonos, M. Zinser, M. Ziolkowski, L. Živković, G. Zobernig, A. Zoccoli, R. Zou, M. zur Nedden, L. Zwalinski, ATLAS Collaboration

**Affiliations:** 1 Department of Physics, University of Adelaide, Adelaide, Australia; 2 Physics Department, SUNY Albany, Albany, NY USA; 3 Department of Physics, University of Alberta, Edmonton, AB Canada; 4 Department of Physics, Ankara University, Ankara, Turkey; 5 Istanbul Aydin University, Istanbul, Turkey; 6 Division of Physics, TOBB University of Economics and Technology, Ankara, Turkey; 7 LAPP, CNRS/IN2P3 and Université Savoie Mont Blanc, Annecy-le-Vieux, France; 8 High Energy Physics Division, Argonne National Laboratory, Argonne, IL USA; 9 Department of Physics, University of Arizona, Tucson, AZ USA; 10 Department of Physics, The University of Texas at Arlington, Arlington, TX USA; 11 Physics Department, National and Kapodistrian University of Athens, Athens, Greece; 12 Physics Department, National Technical University of Athens, Zografou, Greece; 13 Department of Physics, The University of Texas at Austin, Austin, TX USA; 14 Institute of Physics, Azerbaijan Academy of Sciences, Baku, Azerbaijan; 15 Institut de Física d’Altes Energies (IFAE), The Barcelona Institute of Science and Technology, Barcelona, Spain; 16 Institute of Physics, University of Belgrade, Belgrade, Serbia; 17 Department for Physics and Technology, University of Bergen, Bergen, Norway; 18 Physics Division, Lawrence Berkeley National Laboratory, University of California, Berkeley, CA USA; 19 Department of Physics, Humboldt University, Berlin, Germany; 20 Albert Einstein Center for Fundamental Physics, Laboratory for High Energy Physics, University of Bern, Bern, Switzerland; 21 School of Physics and Astronomy, University of Birmingham, Birmingham, UK; 22 Department of Physics, Bogazici University, Istanbul, Turkey; 23 Department of Physics Engineering, Gaziantep University, Gaziantep, Turkey; 24 Faculty of Engineering and Natural Sciences, Istanbul Bilgi University, Istanbul, Turkey; 25 Faculty of Engineering and Natural Sciences, Bahcesehir University, Istanbul, Turkey; 26 Centro de Investigaciones, Universidad Antonio Narino, Bogotá, Colombia; 27 INFN Sezione di Bologna, Bologna, Italy; 28 Dipartimento di Fisica e Astronomia, Università di Bologna, Bologna, Italy; 29 Physikalisches Institut, University of Bonn, Bonn, Germany; 30 Department of Physics, Boston University, Boston, MA USA; 31 Department of Physics, Brandeis University, Waltham, MA USA; 32 Universidade Federal do Rio De Janeiro COPPE/EE/IF, Rio de Janeiro, Brazil; 33 Electrical Circuits Department, Federal University of Juiz de Fora (UFJF), Juiz de Fora, Brazil; 34 Federal University of Sao Joao del Rei (UFSJ), Sao Joao del Rei, Brazil; 35 Instituto de Fisica, Universidade de Sao Paulo, São Paulo, Brazil; 36 Physics Department, Brookhaven National Laboratory, Upton, NY USA; 37 Transilvania University of Brasov, Brasov, Romania; 38 Horia Hulubei National Institute of Physics and Nuclear Engineering, Bucharest, Romania; 39 Department of Physics, Alexandru Ioan Cuza University of Iasi, Iasi, Romania; 40 Physics Department, National Institute for Research and Development of Isotopic and Molecular Technologies, Cluj Napoca, Romania; 41 University Politehnica Bucharest, Bucharest, Romania; 42 West University in Timisoara, Timisoara, Romania; 43 Departamento de Física, Universidad de Buenos Aires, Buenos Aires, Argentina; 44 Cavendish Laboratory, University of Cambridge, Cambridge, UK; 45 Department of Physics, Carleton University, Ottawa, ON Canada; 46 CERN, Geneva, Switzerland; 47 Enrico Fermi Institute, University of Chicago, Chicago, IL USA; 48 Departamento de Física, Pontificia Universidad Católica de Chile, Santiago, Chile; 49 Departamento de Física, Universidad Técnica Federico Santa María, Valparaiso, Chile; 50 Institute of High Energy Physics, Chinese Academy of Sciences, Beijing, China; 51 Department of Physics, Nanjing University, Nanjing, Jiangsu China; 52 Physics Department, Tsinghua University, Beijing, 100084 China; 53 University of Chinese Academy of Science (UCAS), Beijing, China; 54 Department of Modern Physics and State Key Laboratory of Particle Detection and Electronics, University of Science and Technology of China, Anhui, China; 55 School of Physics, Shandong University, Shandong, China; 56 Department of Physics and Astronomy, Key Laboratory for Particle Physics, Astrophysics and Cosmology, Ministry of Education, Shanghai Key Laboratory for Particle Physics and Cosmology, Shanghai Jiao Tong University, Tsung-Dao Lee Institute, Shanghai, China; 57 Université Clermont Auvergne, CNRS/IN2P3, LPC, Clermont-Ferrand, France; 58 Nevis Laboratory, Columbia University, Irvington, NY USA; 59 Niels Bohr Institute, University of Copenhagen, Copenhagen, Denmark; 60 INFN Gruppo Collegato di Cosenza, Laboratori Nazionali di Frascati, Frascati, Italy; 61 Dipartimento di Fisica, Università della Calabria, Rende, Italy; 62 Faculty of Physics and Applied Computer Science, AGH University of Science and Technology, Kraków, Poland; 63 Marian Smoluchowski Institute of Physics, Jagiellonian University, Kraków, Poland; 64 Institute of Nuclear Physics, Polish Academy of Sciences, Kraków, Poland; 65 Physics Department, Southern Methodist University, Dallas, TX USA; 66 Physics Department, University of Texas at Dallas, Richardson, TX USA; 67 DESY, Hamburg and Zeuthen, Germany; 68 Lehrstuhl für Experimentelle Physik IV, Technische Universität Dortmund, Dortmund, Germany; 69 Institut für Kern- und Teilchenphysik, Technische Universität Dresden, Dresden, Germany; 70 Department of Physics, Duke University, Durham, NC USA; 71 SUPA-School of Physics and Astronomy, University of Edinburgh, Edinburgh, UK; 72 INFN e Laboratori Nazionali di Frascati, Frascati, Italy; 73 Fakultät für Mathematik und Physik, Albert-Ludwigs-Universität, Freiburg, Germany; 74 Departement de Physique Nucleaire et Corpusculaire, Université de Genève, Geneva, Switzerland; 75 INFN Sezione di Genova, Genoa, Italy; 76 Dipartimento di Fisica, Università di Genova, Genoa, Italy; 77 E. Andronikashvili Institute of Physics, Iv. Javakhishvili Tbilisi State University, Tbilisi, Georgia; 78 High Energy Physics Institute, Tbilisi State University, Tbilisi, Georgia; 79 II Physikalisches Institut, Justus-Liebig-Universität Giessen, Giessen, Germany; 80 SUPA-School of Physics and Astronomy, University of Glasgow, Glasgow, UK; 81 II Physikalisches Institut, Georg-August-Universität, Göttingen, Germany; 82 Laboratoire de Physique Subatomique et de Cosmologie, Université Grenoble-Alpes, CNRS/IN2P3, Grenoble, France; 83 Laboratory for Particle Physics and Cosmology, Harvard University, Cambridge, MA USA; 84 Kirchhoff-Institut für Physik, Ruprecht-Karls-Universität Heidelberg, Heidelberg, Germany; 85 Physikalisches Institut, Ruprecht-Karls-Universität Heidelberg, Heidelberg, Germany; 86 Faculty of Applied Information Science, Hiroshima Institute of Technology, Hiroshima, Japan; 87 Department of Physics, The Chinese University of Hong Kong, Shatin, NT Hong Kong; 88 Department of Physics, The University of Hong Kong, Hong Kong, China; 89 Department of Physics, Institute for Advanced Study, The Hong Kong University of Science and Technology, Clear Water Bay, Kowloon, Hong Kong, China; 90 Department of Physics, National Tsing Hua University, Taiwan, Taiwan; 91 Department of Physics, Indiana University, Bloomington, IN USA; 92 Institut für Astro- und Teilchenphysik, Leopold-Franzens-Universität, Innsbruck, Austria; 93 University of Iowa, Iowa City, IA USA; 94 Department of Physics and Astronomy, Iowa State University, Ames, IA USA; 95 Joint Institute for Nuclear Research, JINR Dubna, Dubna, Russia; 96 KEK, High Energy Accelerator Research Organization, Tsukuba, Japan; 97 Graduate School of Science, Kobe University, Kobe, Japan; 98 Faculty of Science, Kyoto University, Kyoto, Japan; 99 Kyoto University of Education, Kyoto, Japan; 100 Research Center for Advanced Particle Physics and Department of Physics, Kyushu University, Fukuoka, Japan; 101 Instituto de Física La Plata, Universidad Nacional de La Plata and CONICET, La Plata, Argentina; 102 Physics Department, Lancaster University, Lancaster, UK; 103 INFN Sezione di Lecce, Lecce, Italy; 104 Dipartimento di Matematica e Fisica, Università del Salento, Lecce, Italy; 105 Oliver Lodge Laboratory, University of Liverpool, Liverpool, UK; 106 Department of Experimental Particle Physics, Jožef Stefan Institute and Department of Physics, University of Ljubljana, Ljubljana, Slovenia; 107 School of Physics and Astronomy, Queen Mary University of London, London, UK; 108 Department of Physics, Royal Holloway University of London, Surrey, UK; 109 Department of Physics and Astronomy, University College London, London, UK; 110 Louisiana Tech University, Ruston, LA USA; 111 Laboratoire de Physique Nucléaire et de Hautes Energies, UPMC and Université Paris-Diderot and CNRS/IN2P3, Paris, France; 112 Fysiska institutionen, Lunds universitet, Lund, Sweden; 113 Departamento de Fisica Teorica C-15, Universidad Autonoma de Madrid, Madrid, Spain; 114 Institut für Physik, Universität Mainz, Mainz, Germany; 115 School of Physics and Astronomy, University of Manchester, Manchester, UK; 116 CPPM, Aix-Marseille Université and CNRS/IN2P3, Marseille, France; 117 Department of Physics, University of Massachusetts, Amherst, MA USA; 118 Department of Physics, McGill University, Montreal, QC Canada; 119 School of Physics, University of Melbourne, Victoria, Australia; 120 Department of Physics, The University of Michigan, Ann Arbor, MI USA; 121 Department of Physics and Astronomy, Michigan State University, East Lansing, MI USA; 122 INFN Sezione di Milano, Milan, Italy; 123 Dipartimento di Fisica, Università di Milano, Milan, Italy; 124 B.I. Stepanov Institute of Physics, National Academy of Sciences of Belarus, Minsk, Republic of Belarus; 125 Research Institute for Nuclear Problems of Byelorussian State University, Minsk, Republic of Belarus; 126 Group of Particle Physics, University of Montreal, Montreal, QC Canada; 127 P.N. Lebedev Physical Institute of the Russian Academy of Sciences, Moscow, Russia; 128 Institute for Theoretical and Experimental Physics (ITEP), Moscow, Russia; 129 National Research Nuclear University MEPhI, Moscow, Russia; 130 D.V. Skobeltsyn Institute of Nuclear Physics, M.V. Lomonosov Moscow State University, Moscow, Russia; 131 Fakultät für Physik, Ludwig-Maximilians-Universität München, Munich, Germany; 132 Max-Planck-Institut für Physik (Werner-Heisenberg-Institut), Munich, Germany; 133 Nagasaki Institute of Applied Science, Nagasaki, Japan; 134 Graduate School of Science and Kobayashi-Maskawa Institute, Nagoya University, Nagoya, Japan; 135 INFN Sezione di Napoli, Naples, Italy; 136 Dipartimento di Fisica, Università di Napoli, Naples, Italy; 137 Department of Physics and Astronomy, University of New Mexico, Albuquerque, NM USA; 138 Institute for Mathematics, Astrophysics and Particle Physics, Radboud University Nijmegen/Nikhef, Nijmegen, The Netherlands; 139 Nikhef National Institute for Subatomic Physics, University of Amsterdam, Amsterdam, The Netherlands; 140 Department of Physics, Northern Illinois University, DeKalb, IL USA; 141 Budker Institute of Nuclear Physics, SB RAS, Novosibirsk, Russia; 142 Department of Physics, New York University, New York, NY USA; 143 Ohio State University, Columbus, OH USA; 144 Faculty of Science, Okayama University, Okayama, Japan; 145 Homer L. Dodge Department of Physics and Astronomy, University of Oklahoma, Norman, OK USA; 146 Department of Physics, Oklahoma State University, Stillwater, OK USA; 147 Palacký University, RCPTM, Olomouc, Czech Republic; 148 Center for High Energy Physics, University of Oregon, Eugene, OR USA; 149 LAL, Univ. Paris-Sud, CNRS/IN2P3, Université Paris-Saclay, Orsay, France; 150 Graduate School of Science, Osaka University, Osaka, Japan; 151 Department of Physics, University of Oslo, Oslo, Norway; 152 Department of Physics, Oxford University, Oxford, UK; 153 INFN Sezione di Pavia, Pavia, Italy; 154 Dipartimento di Fisica, Università di Pavia, Pavia, Italy; 155 Department of Physics, University of Pennsylvania, Philadelphia, PA USA; 156 National Research Centre “Kurchatov Institute” B.P. Konstantinov Petersburg Nuclear Physics Institute, St. Petersburg, Russia; 157 INFN Sezione di Pisa, Pisa, Italy; 158 Dipartimento di Fisica E. Fermi, Università di Pisa, Pisa, Italy; 159 Department of Physics and Astronomy, University of Pittsburgh, Pittsburgh, PA USA; 160 Laboratório de Instrumentação e Física Experimental de Partículas-LIP, Lisbon, Portugal; 161 Faculdade de Ciências, Universidade de Lisboa, Lisbon, Portugal; 162 Department of Physics, University of Coimbra, Coimbra, Portugal; 163 Centro de Física Nuclear da Universidade de Lisboa, Lisbon, Portugal; 164 Departamento de Fisica, Universidade do Minho, Braga, Portugal; 165 Departamento de Fisica Teorica y del Cosmos, Universidad de Granada, Granada, Spain; 166 Dep Fisica and CEFITEC of Faculdade de Ciencias e Tecnologia, Universidade Nova de Lisboa, Caparica, Portugal; 167 Institute of Physics, Academy of Sciences of the Czech Republic, Prague, Czech Republic; 168 Czech Technical University in Prague, Prague, Czech Republic; 169 Faculty of Mathematics and Physics, Charles University, Prague, Czech Republic; 170 State Research Center Institute for High Energy Physics (Protvino), NRC KI, Protvino, Russia; 171 Particle Physics Department, Rutherford Appleton Laboratory, Didcot, UK; 172 INFN Sezione di Roma, Rome, Italy; 173 Dipartimento di Fisica, Sapienza Università di Roma, Rome, Italy; 174 INFN Sezione di Roma Tor Vergata, Rome, Italy; 175 Dipartimento di Fisica, Università di Roma Tor Vergata, Rome, Italy; 176 INFN Sezione di Roma Tre, Rome, Italy; 177 Dipartimento di Matematica e Fisica, Università Roma Tre, Rome, Italy; 178 Faculté des Sciences Ain Chock, Réseau Universitaire de Physique des Hautes Energies-Université Hassan II, Casablanca, Morocco; 179 Centre National de l’Energie des Sciences Techniques Nucleaires, Rabat, Morocco; 180 Faculté des Sciences Semlalia, Université Cadi Ayyad, LPHEA-Marrakech, Marrakech, Morocco; 181 Faculté des Sciences, Université Mohamed Premier and LPTPM, Oujda, Morocco; 182 Faculté des Sciences, Université Mohammed V, Rabat, Morocco; 183 DSM/IRFU (Institut de Recherches sur les Lois Fondamentales de l’Univers), CEA Saclay (Commissariat à l’Energie Atomique et aux Energies Alternatives), Gif-sur-Yvette, France; 184 Santa Cruz Institute for Particle Physics, University of California Santa Cruz, Santa Cruz, CA USA; 185 Department of Physics, University of Washington, Seattle, WA USA; 186 Department of Physics and Astronomy, University of Sheffield, Sheffield, UK; 187 Department of Physics, Shinshu University, Nagano, Japan; 188 Department Physik, Universität Siegen, Siegen, Germany; 189 Department of Physics, Simon Fraser University, Burnaby, BC Canada; 190 SLAC National Accelerator Laboratory, Stanford, CA USA; 191 Faculty of Mathematics, Physics and Informatics, Comenius University, Bratislava, Slovak Republic; 192 Department of Subnuclear Physics, Institute of Experimental Physics of the Slovak Academy of Sciences, Kosice, Slovak Republic; 193 Department of Physics, University of Cape Town, Cape Town, South Africa; 194 Department of Physics, University of Johannesburg, Johannesburg, South Africa; 195 School of Physics, University of the Witwatersrand, Johannesburg, South Africa; 196 Department of Physics, Stockholm University, Stockholm, Sweden; 197 The Oskar Klein Centre, Stockholm, Sweden; 198 Physics Department, Royal Institute of Technology, Stockholm, Sweden; 199 Departments of Physics and Astronomy and Chemistry, Stony Brook University, Stony Brook, NY USA; 200 Department of Physics and Astronomy, University of Sussex, Brighton, UK; 201 School of Physics, University of Sydney, Sydney, Australia; 202 Institute of Physics, Academia Sinica, Taipei, Taiwan; 203 Department of Physics, Technion: Israel Institute of Technology, Haifa, Israel; 204 Raymond and Beverly Sackler School of Physics and Astronomy, Tel Aviv University, Tel Aviv, Israel; 205 Department of Physics, Aristotle University of Thessaloniki, Thessaloniki, Greece; 206 International Center for Elementary Particle Physics and Department of Physics, The University of Tokyo, Tokyo, Japan; 207 Graduate School of Science and Technology, Tokyo Metropolitan University, Tokyo, Japan; 208 Department of Physics, Tokyo Institute of Technology, Tokyo, Japan; 209 Tomsk State University, Tomsk, Russia; 210 Department of Physics, University of Toronto, Toronto, ON Canada; 211 INFN-TIFPA, Trento, Italy; 212 University of Trento, Trento, Italy; 213 TRIUMF, Vancouver, BC Canada; 214 Department of Physics and Astronomy, York University, Toronto, ON Canada; 215 Faculty of Pure and Applied Sciences, and Center for Integrated Research in Fundamental Science and Engineering, University of Tsukuba, Tsukuba, Japan; 216 Department of Physics and Astronomy, Tufts University, Medford, MA USA; 217 Department of Physics and Astronomy, University of California Irvine, Irvine, CA USA; 218 INFN Gruppo Collegato di Udine, Sezione di Trieste, Udine, Italy; 219 ICTP, Trieste, Italy; 220 Dipartimento di Chimica, Fisica e Ambiente, Università di Udine, Udine, Italy; 221 Department of Physics and Astronomy, University of Uppsala, Uppsala, Sweden; 222 Department of Physics, University of Illinois, Urbana, IL USA; 223 Instituto de Fisica Corpuscular (IFIC), Centro Mixto Universidad de Valencia-CSIC, Valencia, Spain; 224 Department of Physics, University of British Columbia, Vancouver, BC Canada; 225 Department of Physics and Astronomy, University of Victoria, Victoria, BC Canada; 226 Department of Physics, University of Warwick, Coventry, UK; 227 Waseda University, Tokyo, Japan; 228 Department of Particle Physics, The Weizmann Institute of Science, Rehovot, Israel; 229 Department of Physics, University of Wisconsin, Madison, WI USA; 230 Fakultät für Physik und Astronomie, Julius-Maximilians-Universität, Würzburg, Germany; 231 Fakultät für Mathematik und Naturwissenschaften, Fachgruppe Physik, Bergische Universität Wuppertal, Wuppertal, Germany; 232 Department of Physics, Yale University, New Haven, CT USA; 233 Yerevan Physics Institute, Yerevan, Armenia; 234 Centre de Calcul de l’Institut National de Physique Nucléaire et de Physique des Particules (IN2P3), Villeurbanne, France; 235 Academia Sinica Grid Computing, Institute of Physics, Academia Sinica, Taipei, Taiwan; 236 CERN, 1211 Geneva 23, Switzerland

## Abstract

The differential cross-section for the production of a *W* boson in association with a top quark is measured for several particle-level observables. The measurements are performed using ${36.1}\,\text {fb}^{-1}$ of *pp* collision data collected with the ATLAS detector at the LHC in 2015 and 2016. Differential cross-sections are measured in a fiducial phase space defined by the presence of two charged leptons and exactly one jet matched to a *b*-hadron, and are normalised with the fiducial cross-section. Results are found to be in good agreement with predictions from several Monte Carlo event generators.

## Introduction

**Fig. 1:**
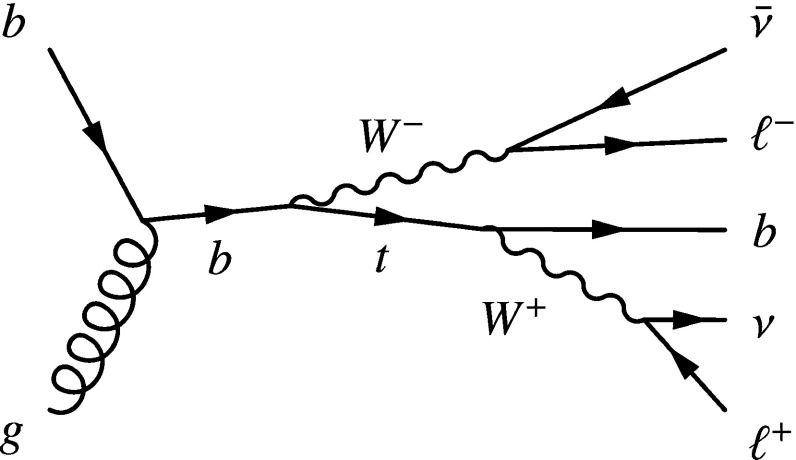
A representative leading-order Feynman diagram for the production of a single top quark in the *tW* channel and the subsequent leptonic decay of the *W* boson and semileptonic decay of the top quark

Single-top-quark production proceeds via three channels through electroweak interactions involving a *Wtb* vertex at leading order (LO) in the Standard Model (SM): the *t*-channel, the *s*-channel, and production in association with a *W* boson (*tW*). The cross-section for each of these channels depends on the relevant Cabibbo–Kobayashi–Maskawa (CKM) matrix element $V_{tb}$ and form factor $f^\mathrm {L}_\mathrm {V}$ [1–3] such that the cross-section is proportional to $|f^\mathrm {L}_\mathrm {V}V_{tb}|^{2}$ [4, 5], i.e. depends on the coupling between the *W* boson, top and *b* quarks. The *tW* channel, represented in Fig. 1, has a *pp* production cross-section at $\sqrt{s} ={13} \hbox { TeV}$ of ${\sigma _{\text {theory}}= {71.7\pm 1.8\,(\mathrm {scale})\pm {3.4}\,(\mathrm {PDF})}\,{\mathrm {pb}}}$ [6], and contributes approximately $24\%$ of the total single-top-quark production rate at 13 TeV. At the LHC, evidence for this process with 7 TeV collision data was presented by the ATLAS Collaboration [7] (with a significance of $3.6\sigma$), and by the CMS Collaboration [8] (with a significance of $4.0\sigma$). With 8 TeV collision data, CMS observed the *tW* channel with a significance of $6.1\sigma$ [9] while ATLAS observed it with a significance of $7.7\sigma$ [10]. This analysis extends an ATLAS analysis [11] which measured the production cross-section with 13 TeV data collected in 2015.

Accurate estimates of rates and kinematic distributions of the *tW* process are difficult at higher orders in $\alpha _{\text {S}}$ since the process is not well-defined due to quantum interference with the $t\bar{t}$ production process. A fully consistent theoretical picture can be reached by considering *tW* and $t\bar{t}$ to be components of the complete *WbWb* final state in the four flavour scheme [12]. In the $t\bar{t}$ process the two *Wb* systems are produced on the top quark mass shell, and so a proper treatment of this doubly resonant component is important in the study of *tW* beyond leading order. Two commonly used approaches are diagram removal (DR) and diagram subtraction (DS) [13]. In the DR approach, all next-to-leading order (NLO) diagrams that overlap with the doubly resonant $t\bar{t}$ contributions are removed from the calculation of the *tW* amplitude, violating gauge invariance. In the DS approach, a subtraction term is built into the amplitude to cancel out the $t\bar{t}$ component close to the top quark resonance while respecting gauge invariance.

This paper describes differential cross-section measurements in the *tW* dilepton final state, where events contain two oppositely charged leptons (henceforth “lepton” refers to an electron or muon) and two neutrinos. This channel is chosen because it has a better ratio of signal and $t\bar{t}$ production over other background processes than the single lepton+jets channel, where large $W+$jets backgrounds are relatively difficult to separate from top quark events. Distributions are unfolded to observables based on stable particles produced in Monte Carlo (MC) simulation. Measurements are performed in a fiducial phase space, defined by the presence of two charged leptons as well as the presence of exactly one central jet containing *b*-hadrons (*b*-jet) and no other jets. This requirement on the jet multiplicity is expected to suppress the contribution from $t\bar{t}$ production, where a pair of *b*-jets is more commonly produced, as well as reducing the importance of $t\bar{t}$-*tW* interference effects [12]. After applying the reconstruction-level selection of fiducial events (described in Sect. 5) backgrounds from $t\bar{t}$ and other sources are subtracted according to their predicted distributions from MC simulation. The definition of the fiducial event selection is chosen to match the lepton and jet requirements at reconstruction level. Exactly two leptons with $p_{\text {T}} >20 \hbox { GeV}$ and $|\eta |<2.5$ are required, and at least one of the leptons must satisfy $p_{\text {T}} >{27} \hbox { GeV}$. Exactly one *b*-tagged jet satisfying $p_{\text {T}} >25 \hbox { GeV}$ and $|\eta |<2.5$ must be present. No requirement is placed on $E_{\text {T}}^{\text {miss}}$ or $m_{\ell \ell }$. A boosted decision tree (BDT) is used to separate the *tW* signal from the large $t\bar{t}$ background by placing a fixed requirement on the BDT response.

Although the top quark and the two *W* bosons cannot be directly reconstructed due to insufficient kinematic constraints, one can select a list of observables that are correlated with kinematic properties of *tW* production and are sensitive to differences in theoretical modelling. Particle energies and masses are also preferred to projections onto the transverse plane in order to be sensitive to polar angular information while keeping the list of observables as short as possible. Unfolded distributions are measured for:

- the energy of the *b*-jet, $E(b)$;
- the mass of the leading lepton and *b*-jet, $m(\ell _1 b)$;
- the mass of the sub-leading lepton and the *b*-jet, $m(\ell _2 b)$;
- the energy of the system of the two leptons and *b*-jet, $E(\ell \ell b)$;
- the transverse mass of the leptons, *b*-jet and neutrinos, $m_{\text {T}} (\ell \ell \nu \nu b)$; and
- the mass of the two leptons and the *b*-jet, $m(\ell \ell b)$.

The top quark production is probed most directly by $E(b)$, the only final-state object that can unambiguously be matched to the decay products of the top quark. The top-quark decay is probed by $m(\ell _1 b)$ and $m(\ell _2 b)$, which are sensitive to angular correlations of decay products due to production spin correlations. The combined *tW*-system is probed by $E(\ell \ell b)$, $m_{\text {T}} (\ell \ell \nu \nu b)$, and $m(\ell \ell b)$. At reconstruction level, the transverse momenta of the neutrinos in $m_{\text {T}} (\ell \ell \nu \nu b)$ are represented by the measured $E_{\text {T}}^{\text {miss}}$ (reconstructed as described in Sect. 4). At particle level the vector summed transverse momenta of simulated neutrinos (selected as defined in Sect. 4) are used in $m_{\text {T}} (\ell \ell \nu \nu b)$. All other quantities for leptons and jets are taken simply from the relevant reconstructed or particle-level objects. These observables are selected to minimise the bias introduced by the BDT requirement, as certain observables are highly correlated with the BDT discriminant. These cannot be effectively unfolded due to shaping effects that the BDT requirement imposes on the overall acceptance, and thus are not considered in this measurement. The background-subtracted data are unfolded using an iterative procedure [14] to correct for resolution and acceptance effects, biases, and particles outside the fiducial phase space of the measurement. The differential cross-sections are normalised with the fiducial cross-section, which cancels out many of the largest uncertainties.

## ATLAS detector

The ATLAS detector [15] at the LHC covers nearly the entire solid angle^1^ around the collision point, and consists of an inner tracking detector (ID) surrounded by a thin superconducting solenoid producing a 2 T axial magnetic field, electromagnetic (EM) and hadronic calorimeters, and an external muon spectrometer (MS). The ID consists of a high-granularity silicon pixel detector and a silicon microstrip tracker, together providing precision tracking in the pseudorapidity range $|\eta |<2.5$, complemented by a transition radiation tracker providing tracking and electron identification information for $|\eta |<2.0$. The innermost pixel layer, the insertable B-layer [16], was added between Run 1 and Run 2 of the LHC, at a radius of $33\hbox { mm}$ around a new, thinner, beam pipe. A lead liquid-argon (LAr) electromagnetic calorimeter covers the region $|\eta |<3.2$, and hadronic calorimetry is provided by steel/scintillator tile calorimeters within $|\eta |<1.7$ and copper/LAr hadronic endcap calorimeters in the range $1.5< |\eta |< 3.2$. A LAr forward calorimeter with copper and tungsten absorbers covers the range $3.1< |\eta | <4.9$. The MS consists of precision tracking chambers covering the region $|\eta |<2.7$, and separate trigger chambers covering $|\eta |<2.4$. A two-level trigger system [17], using a custom hardware level followed by a software-based level, selects from the 40 MHz of collisions a maximum of around 1 kHz of events for offline storage.

## Data and Monte Carlo samples

The data events analysed in this paper correspond to an integrated luminosity of $36.1~\hbox {fb}^{-1}$ collected from the operation of the LHC in 2015 and 2016 at $\sqrt{s} ={13} \hbox { TeV}$ with a bunch spacing of 25 ns and an average number of collisions per bunch crossing $\langle \mu \rangle$ of around 23. They are required to be recorded in periods where all detector systems are flagged as operating normally.

Monte Carlo simulated samples are used to estimate the efficiency to select signal and background events, train and test the BDT, estimate the migration of observables from particle level to reconstruction level, estimate systematic uncertainties, and validate the analysis tools. The nominal samples, used for estimating the central values for efficiencies and background templates, were simulated with a full ATLAS detector simulation [18] implemented in Geant  4 [19]. Many of the samples used in the estimation of systematic uncertainties were instead produced using Atlfast2 [20], in which a parameterised detector simulation is used for the calorimeter responses. Pile-up (additional *pp* collisions in the same or a nearby bunch crossing) is included in the simulation by overlaying collisions with the soft QCD processes from Pythia  8.186 [21] using a set of tuned parameters called the A2 tune [22] and the MSTW2008LO parton distribution function (PDF) set [23]. Events were generated with a predefined distribution of the expected number of interactions per bunch crossing, then reweighted to match the actual observed data conditions. In all MC samples and fixed-order calculations used for this analysis the top quark mass $m_{\text {t}}$ is set to 172.5 GeV and the $W\rightarrow \ell \nu$ branching ratio is set to 0.108 per lepton flavour. The EvtGen v1.2.0 program [24] was used to simulate properties of the bottom and charmed hadron decays except for samples generated with Sherpa, which uses internal modules.

The nominal *tW* event samples [25] were produced using the Powheg-Box  v1 [26–30] event generator with the CT10 PDF set [31] in the matrix-element calculations. The parton shower, hadronisation, and underlying event were simulated using Pythia 6.428  [32] with the CTEQ6L1 PDF set [33] and the corresponding Perugia 2012 (P2012) tune [34]. The DR scheme [13] was employed to handle the interference between *tW* and $t\bar{t}$, and was applied to the *tW* sample. For comparing MC predictions to data, the predicted *tW* cross-section at $\sqrt{s} ={13}\hbox { TeV}$ is scaled by a *K*-factor and set to the NLO value with next-to-next-to-leading logarithmic (NNLL) soft-gluon corrections: ${\sigma _{\text {theory}}= {{71.7\pm 1.8\,(\mathrm {scale})\pm {3.4}\,(\mathrm {PDF})}\,{\mathrm {pb}}}}$ [6]. The first uncertainty accounts for the renormalisation and factorisation scale variations (from 0.5 to 2 times $m_{\text {t}}$), while the second uncertainty originates from uncertainties in the MSTW2008 NLO PDF sets.

Additional *tW* samples were generated to estimate systematic uncertainties in the modelling of the signal process. An alternative *tW* sample was generated using the DS scheme instead of DR. A *tW* sample generated with MadGraph5_aMC@NLO  v2.2.2 [35] (instead of the Powheg-Box) interfaced with Herwig++  2.7.1 [36] and processed through the Atlfast2 fast simulation is used to estimate uncertainties associated with the modelling of the NLO matrix-element event generator. A sample generated with Powheg-Box interfaced with Herwig++ (instead of Pythia 6) is used to estimate uncertainties associated with the parton shower, hadronisation, and underlying-event models. This sample is also compared with the previously mentioned MadGraph5_aMC@NLO sample to estimate a matrix-element event generator uncertainty with a consistent parton shower event generator. In both cases, the UE-EE-5 tune of Ref. [37] was used for the underlying event. Finally, in order to estimate uncertainties arising from additional QCD radiation in the *tW* events, a pair of samples were generated with Powheg-Box interfaced with Pythia 6 using Atlfast2 and the P2012 tune with higher and lower radiation relative to the nominal set, together with varied renormalisation and factorisation scales. In order to avoid comparing two different detector response models when estimating systematic uncertainties, another version of the nominal Powheg-Box with Pythia 6 sample was also produced with Atlfast2.

The nominal $t\bar{t}$ event sample [25] was produced using the Powheg-Box  v2 [26–30] event generator with the CT10 PDF set [31] in the matrix-element calculations. The parton shower, hadronisation, and underlying event were simulated using Pythia 6.428  [32] with the CTEQ6L1 PDF set [33] and the corresponding Perugia 2012 (P2012) tune [34]. The renormalisation and factorisation scales are set to $m_{\text {t}}$ for the *tW* process and to $\sqrt{m_{\text {t}}^2 + {p_{\text {T}} (t)}^2}$ for the $t\bar{t}$ process, and the $h_\text {damp}$ resummation damping factor is set to equal the mass of the top quark.

Additional $t\bar{t}$ samples were generated to estimate systematic uncertainties. Like the additional *tW* samples, these are used to estimate the uncertainties associated with the matrix-element event generator (a sample produced using Atlfast2 fast simulation with MadGraph5_aMC@NLO  v2.2.2 interfaced with Herwig++  2.7.1), parton shower and hadronisation models (a sample produced using Atlfast2 with Powheg-Box interfaced with Herwig++  2.7.1) and additional QCD radiation. To estimate uncertainties on additional QCD radiation in $t\bar{t}$, a pair of samples is produced using full simulation with the varied sets of P2012 parameters for higher and lower radiation, as well as with varied renormalisation and factorisation scales. In these samples the resummation damping factor $h_\text {damp}$ is doubled in the case of higher radiation. The $t\bar{t}$ cross-section is set to $\sigma _{t\bar{t}} = {831.8\,^{+19.8}_{-29.2}\,(\mathrm {scale})\,\pm 35.1\,(\mathrm {PDF} + \alpha _{\text {S}})}\,{\hbox {pb}}$ as calculated with the Top++ 2.0 program to NNLO, including soft-gluon resummation to NNLL [38]. The first uncertainty comes from the independent variation of the factorisation and renormalisation scales, $\mu _{\mathrm {F}}$ and $\mu _{\mathrm {R}}$, while the second one is associated with variations in the PDF and $\alpha _{\text {S}}$, following the PDF4LHC prescription with the MSTW2008 $68\%$ CL NNLO, CT10 NNLO and NNPDF2.3 5f FFN PDF sets [39–42].

Samples used to model the $Z {\,\text {+}\,\text {jets}}$ background [43] were simulated with Sherpa 2.2.1  [44]. In these, the matrix element is calculated for up to two partons at NLO and four partons at LO using Comix [45] and OpenLoops [46], and merged with the Sherpa parton shower [47] using the ME+PS@NLO prescription [48]. The NNPDF3.0 NNLO PDF set [49] was used in conjunction with Sherpa parton shower tuning, with a generator-level cut-off on the dilepton invariant mass of $m_{\ell \ell } >40~\hbox {GeV}$ applied. The $Z {\,\text {+}\,\text {jets}}$ events are normalised using NNLO cross-sections computed with FEWZ [50].

Diboson processes with four charged leptons, three charged leptons and one neutrino, or two charged leptons and two neutrinos [51] were simulated using the Sherpa 2.1.1 event generator. The matrix elements contain all diagrams with four electroweak vertices. NLO calculations were used for the purely leptonic final states as well as for final states with two or four charged leptons plus one additional parton. For other final states with up to three additional partons, the LO calculations of Comix and OpenLoops were used. Their outputs were combined with the Sherpa parton shower using the ME+PS@NLO prescription [48]. The CT10 PDF set with dedicated parton shower tuning was used. The cross-sections provided by the event generator (which are already at NLO) were used for diboson processes.

## Object reconstruction

Electron candidates are reconstructed from energy deposits in the EM calorimeter associated with ID tracks [17]. The deposits are required to be in the $|\eta |<2.47$ region, with the transition region between the barrel and endcap EM calorimeters, $1.37<|\eta |<1.52$, excluded. The candidate electrons are required to have a transverse momentum of $p_{\text {T}} >20~\hbox {GeV}$. Further requirements on the electromagnetic shower shape, ratio of calorimeter energy to tracker momentum, and other variables are combined into a likelihood-based discriminant [52], with signal electron efficiencies measured to be at least 85%, increasing for higher $p_{\text {T}}$. Candidate electrons also must satisfy requirements on the distance from the ID track to the beamline or to the reconstructed primary vertex in the event, which is identified as the vertex with the largest summed $p_{\text {T}} ^2$ of associated tracks. The transverse impact parameter with respect to the beamline, $d_0$, must satisfy $|d_0|/\sigma _{d_{0}} < 5$, where $\sigma _{d_0}$ is the uncertainty in $d_0$. The longitudinal impact parameter, $z_0$, must satisfy $|\Delta z_0 \sin \theta |<0.5~\hbox {mm}$, where $\Delta z_0$ is the longitudinal distance from the primary vertex along the beamline and $\theta$ is the angle of the track to the beamline. Furthermore, electrons must satisfy isolation requirements based on ID tracks and topological clusters in the calorimeter [53], designed to achieve an isolation efficiency of $90\%$ ($99\%$) for $p_{\text {T}} = {25(60)}~\hbox {GeV}$.

Muon candidates are identified by matching MS tracks with ID tracks [54]. The candidates must satisfy requirements on hits in the MS and on the compatibility of ID and MS momentum measurements to remove fake muon signatures. Furthermore, they must have $p_{\text {T}} >20 \hbox { GeV}$ as well as $|\eta |<2.5$ to ensure they are within coverage of the ID. Candidate muons must satisfy the following requirements on the distance from the combined ID and MS track to the beamline or primary vertex: the transverse impact parameter significance must satisfy $|d_0|/\sigma _{d_{0}} < 3$, and the longitudinal impact parameter must satisfy $|\Delta z_0 \sin \theta |<0.5~\hbox {mm}$, where $d_0$ and $z_0$ are defined as above for electrons. An isolation requirement based on ID tracks and topological clusters in the calorimeter is imposed, which targets an isolation efficiency of $90\%$ ($99\%$) for $p_{\text {T}} = {25(60)}~\hbox {GeV}$.

Jets are reconstructed from topological clusters of energy deposited in the calorimeter [53] using the anti-$k_t$ algorithm [55] with a radius parameter of 0.4 implemented in the FastJet package [56]. Their energies are corrected to account for pile-up and calibrated using a $p_{\text {T}}$- and $\eta$-dependent correction derived from Run 2 data [57]. They are required to have $p_{\text {T}} >25~\hbox {GeV}$ and $|\eta |<2.5$. To suppress pile-up, a discriminant called the jet-vertex-tagger is constructed using a two-dimensional likelihood method [58]. For jets with $p_{\text {T}} <60~\hbox {GeV}$ and $|\eta | < 2.4$, a jet-vertex-tagger requirement corresponding to a $92\%$ efficiency while rejecting $98\%$ of jets from pile-up and noise is imposed.

The tagging of *b*-jets uses a multivariate discriminant which exploits the long lifetime of *b*-hadrons and large invariant mass of their decay products relative to *c*-hadrons and unstable light hadrons [59, 60]. The discriminant is calibrated to achieve a $77\%$ $b\text {-tagging}$ efficiency and a rejection factor of about 4.5 against jets containing charm quarks (*c*-jets) and 140 against light-quark and gluon jets in a sample of simulated $t\bar{t}$ events. The jet tagging efficiency in simulation is corrected to the efficiency in data [61].

The missing transverse momentum vector is calculated as the negative vectorial sum of the transverse momenta of particles in the event. Its magnitude, $E_{\text {T}}^{\text {miss}}$, is a measure of the transverse momentum imbalance, primarily due to neutrinos that escape detection. In addition to the identified jets, electrons and muons, a track-based soft term is included in the $E_{\text {T}}^{\text {miss}}$ calculation by considering tracks associated with the hard-scattering vertex in the event which are not also associated with an identified jet, electron, or muon [62, 63].

To avoid cases where the detector response to a single physical object is reconstructed as two separate final-state objects, several steps are followed to remove such overlaps. First, identified muons that deposit energy in the calorimeter and share a track with an electron are removed, followed by the removal of any remaining electrons sharing a track with a muon. This step is designed to avoid cases where a muon mimics an electron through radiation of a hard photon. Next, the jet closest to each electron within a *y*–$\phi$ cone of size $\Delta R_{y,\phi } \equiv \sqrt{{(\Delta y)}^2 + {(\Delta \phi )}^2} = 0.2$ is removed to reduce the proportion of electrons being reconstructed as jets. Next, electrons with a distance $\Delta R_{y,\phi } < 0.4$ from any of the remaining jets are removed to reduce backgrounds from non-prompt, non-isolated electrons originating from heavy-flavour hadron decays. Jets with fewer than three tracks and distance $\Delta R_{y,\phi } < 0.2$ from a muon are then removed to reduce the number of jet fakes from muons depositing energy in the calorimeters. Finally, muons with a distance $\Delta R_{y,\phi } < 0.4$ from any of the surviving jets are removed to avoid contamination due to non-prompt muons from heavy-flavour hadron decays.

Definitions of particle-level objects in MC simulation are based on stable ($c \tau >10~\hbox {mm}$) outgoing particles [64]. Particle-level prompt charged leptons and neutrinos that arise from decays of *W* bosons or *Z* bosons are accepted. The charged leptons are then dressed with nearby photons, considering all photons that satisfy $\Delta R_{y,\phi } (\ell ,\gamma ) < 0.1$ and do not originate from hadrons, adding the four-momenta of all selected photons to the bare lepton to obtain the dressed lepton four-momentum. Particle-level jets are built from all remaining stable particles in the event after excluding leptons and the photons used to dress the leptons, clustering them using the anti-$k_t$ algorithm with $R=0.4$. Particle-level jet *b*-tagging is performed by checking the jets for any associated *b*-hadron with $p_{\text {T}} >5~\hbox {GeV}$. This association is achieved by reclustering jets with *b*-hadrons included in the input list of particles, but with their $p_{\text {T}}$ scaled down to negligibly small values. Jets containing *b*-hadrons after this reclustering are considered to be associated to a *b*-hadron.

## Event selection

Events passing the reconstruction-level selection are required to have at least one interaction vertex, to pass a single-electron or single-muon trigger, and to contain at least one jet with $p_{\text {T}} >25~\hbox {GeV}$. Single-lepton triggers used in this analysis are designed to select events containing a well-identified charged lepton with high transverse momentum [17]. They require a $p_{\text {T}}$ of at least 20 GeV (26 GeV) for muons and 24 GeV (26 GeV) for electrons for the 2015 (2016) data set, and also have requirements on the lepton quality and isolation. These are complemented by triggers with higher $p_{\text {T}}$ thresholds and relaxed isolation and identification requirements to ensure maximum efficiency at higher lepton $p_{\text {T}}$.

Events are required to contain exactly two oppositely charged leptons with $p_{\text {T}} >20~\hbox {GeV}$; events with a third charged lepton with $p_{\text {T}} >20~\hbox {GeV}$ are rejected. At least one lepton must have $p_{\text {T}} >27~\hbox {GeV}$, and at least one of the selected electrons (muons) must be matched within a $\Delta R_{y,\phi }$ cone of size 0.07 (0.1) to the electron (muon) selected online by the corresponding trigger.

In simulated events, information recorded by the event generator is used to identify events in which any selected lepton does not originate promptly from the hard-scatter process. These non-prompt or fake leptons arise from processes such as the decay of a heavy-flavour hadron, photon conversion or hadron misidentification, and are identified when the electron or muon does not originate from the decay of a *W* or *Z* boson (or a $\tau$ lepton itself originating from a *W* or *Z*). Events with a selected lepton which is non-prompt or fake are themselves labelled as fake and, regardless of whether they are *tW* fake events or fake events from other sources, they are treated as a contribution to the background.

After this selection has been made, a further set of requirements is imposed with the aim of reducing the contribution from the $Z {\,\text {+}\,\text {jets}}$, diboson and fake-lepton backgrounds. The samples consist almost entirely of *tW* signal and $t\bar{t}$ background, which are subsequently separated by the BDT discriminant. Events in which the two leptons have the same flavour and an invariant mass consistent with a *Z* boson ($81<m_{\ell \ell }<101~\hbox {GeV}$) are vetoed, as well as those with an invariant mass $m_{\ell \ell }<40~\hbox {GeV}$. Further requirements placed on $E_{\text {T}}^{\text {miss}}$ and $m_{\ell \ell }$ depend on the flavour of the selected leptons. Events with different-flavour leptons contain backgrounds from $Z\rightarrow \tau \tau$, and are required to have $E_{\text {T}}^{\text {miss}} >20~\hbox {GeV}$, with the requirement raised to $E_{\text {T}}^{\text {miss}} >50~\hbox {GeV}$ when the dilepton invariant mass satisfies $m_{\ell \ell } <80~\hbox {GeV}$. All events with same-flavour leptons, which contain backgrounds from $Z\rightarrow ee$ and $Z\rightarrow \mu \mu$, must satisfy $E_{\text {T}}^{\text {miss}} >40~\hbox {GeV}$. For same-flavour leptons, the $Z {\,\text {+}\,\text {jets}}$ background is concentrated in a region of the $m_{\ell \ell }$–$E_{\text {T}}^{\text {miss}}$ plane corresponding to values of $m_{\ell \ell }$ near the *Z* mass, and towards low values of $E_{\text {T}}^{\text {miss}}$. Therefore, a selection in $E_{\text {T}}^{\text {miss}}$ and $m_{\ell \ell }$ is used to remove these backgrounds: events with $40~\hbox {GeV}<m_{\ell \ell } <81~\hbox {GeV}$ are required to satisfy $E_{\text {T}}^{\text {miss}} {} > 1.25\times m_{\ell \ell }$ while events with $m_{\ell \ell } >101~\hbox {GeV}$ are required to satisfy $E_{\text {T}}^{\text {miss}} >300~\hbox {GeV} - 2\times m_{\ell \ell }$.

**Fig. 2:**
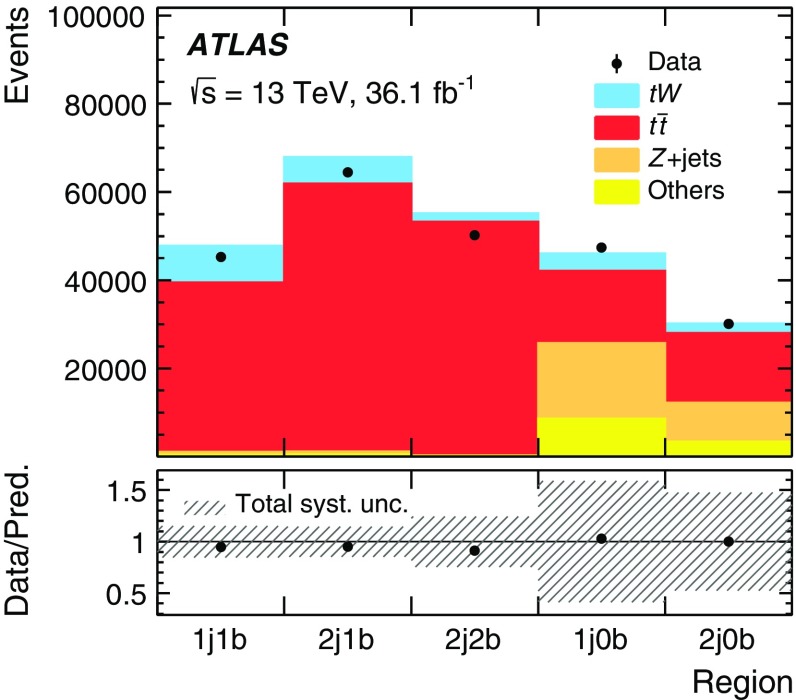
Expected event yields for signal and backgrounds with their total systematic uncertainty (discussed in Sect. 8) and the number of observed events in data shown in the signal region (labelled 1j1b) and the four additional regions (labelled 2j1b, 2j2b, 1j0b and 2j0b, based on the number of selected jets and *b*-tagged jets). “Others” includes diboson and fake-lepton backgrounds. The signal and backgrounds are normalised to their theoretical predictions, and the error bands in the lower panel represent the total systematic uncertainties which are used in this analysis. The upper panel gives the yields in number of events per bin, while the lower panel gives the ratios of the numbers of observed events to the total prediction in each bin

**Fig. 3:**
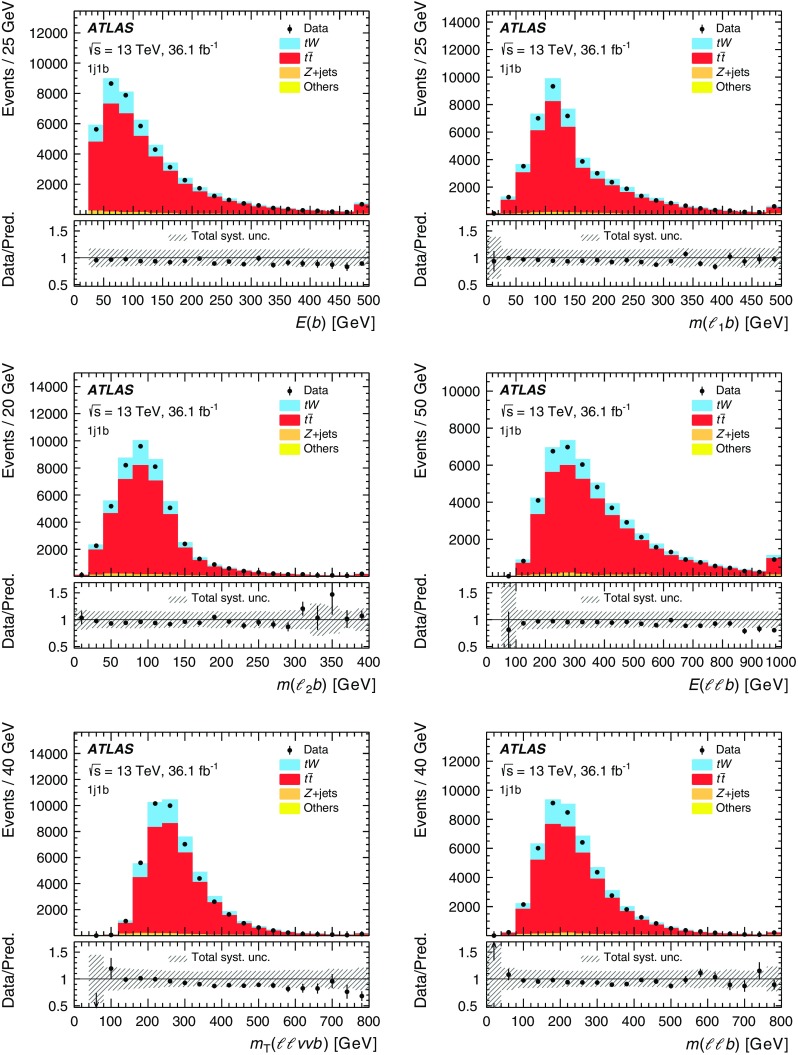
Distributions of the observables chosen to be unfolded after selection at the reconstruction level but before applying the BDT selection. The signal and backgrounds are normalised to their theoretical predictions, and the error bands represent the total systematic uncertainties in the MC predictions. The last bin of each distribution contains overflow events. The panels give the yields in number of events, and the ratios of the numbers of observed events to the total prediction in each bin

Finally, events are required to have exactly one jet which is *b*-tagged. For validation of the signal and background models, additional regions are also defined according to the number of jets and the number of *b*-tagged jets, but are not used in the differential cross-section measurement, primarily due to the lower signal purity in these regions. These regions are labelled by the number *n* of selected jets and the number *m* of selected *b*-tagged jets as *n*j*m*b (for example the 2j1b region consists of events with 2 selected jets of which 1 is *b*-tagged), and show good agreement between data and predictions. The event yields for signal and backgrounds with their total systematic uncertainties, as well as the number of observed events in the data in the signal and validation regions are shown in Fig. 2, and the yields in the signal region are shown in Table 1. Distributions of the events passing these requirements are shown in Fig. 3 at reconstruction level. Most of the predictions agree well with data within the systematic errors, which are highly correlated bin-to-bin due to the dominance of a small number of sources of large normalisation uncertainties. The distribution of $m_{\text {T}} (\ell \ell \nu \nu b)$ , which shows a slope in the ratio of data to prediction, has a *p* value of 2–4% for the predictions to describe the observed distribution after taking bin-to-bin correlations into account.

**Table 1:** Predicted and observed yields in the 1j1b signal region before and after the application of the BDT requirement

Process	Events	Events BDT response $> 0.3$
*tW*	$8300 \pm 1400$	$1970 \pm 560$
$t\bar{t}$	$38{,}400 \pm 6600$	$3400 \pm 1300$
$Z {\,\text {+}\,\text {jets}}$	$620 \pm 310$	$159 \pm 80$
Diboson	$230 \pm 58$	$81 \pm 20$
Fakes	$220 \pm 220$	$19 \pm 19$
Predicted	$47{,}800 \pm 7300$	$5600 \pm 1700$
Observed	45,273	5043

## Separation of *tW* signal from $t\bar{t}$ background

To separate *tW* signal events from background $t\bar{t}$ events, a BDT technique [65] is used to combine several observables into a single discriminant. In this analysis, the BDT implementation is provided by the TMVA package [66], using the GradientBoost algorithm. The approach is based on the BDT developed for the inclusive cross-section measurement in Ref. [11].

The BDT is optimised by using the sum of the nominal *tW* MC sample, the alternative *tW* MC sample with the diagram subtraction scheme and the nominal $t\bar{t}$ MC sample; for each sample, half of the events are used for training while the other half is reserved for testing. A large list of variables is prepared to serve as inputs to the BDT. An optimisation procedure is then carried out to select a subset of input variables and a set of BDT parameters (such as the number of trees in the ensemble and the maximum depth of the individual decision trees). The optimisation is designed to provide the best separation between the *tW* signal and the $t\bar{t}$ background while avoiding sensitivity to statistical fluctuations in the training sample.

The variables considered are derived from the kinematic properties of subsets of the selected physics objects defined in Sect. 4 for each event. For a set of objects $o_1 \cdots o_n$: $p_{\text {T}} (o_1 \cdots o_n)$ is the transverse momentum of vector sums of various subsets; $\sum {E_{\text {T}}}$ is the scalar sum of the transverse momenta of all objects which contribute to the $E_{\text {T}}^{\text {miss}}$ calculation; $\eta (o_1 \cdots o_n)$ is the pseudorapidity of vector sums of various subsets; $m(o_1 \cdots o_n)$ is the invariant mass of various subsets. For vector sums of two systems of objects $s_{1}$ and $s_{2}$: $\Delta p_{\text {T}} (s_1, s_2)$ is the $p_{\text {T}}$ difference; and $C(s_1 s_2)$ is the ratio of the scalar sum of $p_{\text {T}}$ to the sum of energy, called the centrality.

**Fig. 4:**
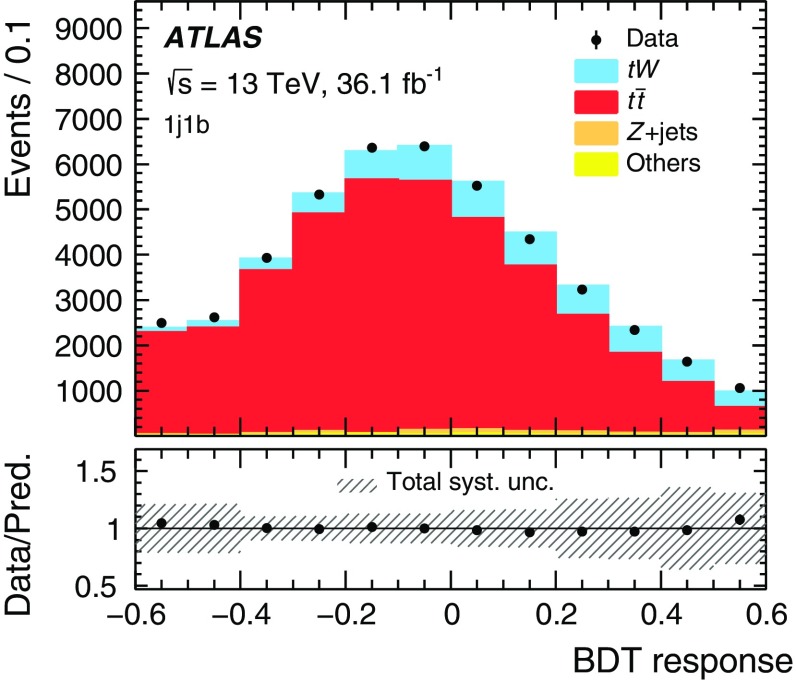
Comparison of data and MC predictions for the BDT response in the signal region. The *tW* signal is normalised with the measured fiducial cross-section. Uncertainty bands reflect the total systematic uncertainties. The first and last bins contain underflow and overflow events, respectively

The final set of input variables used in the BDT is listed in Table 2 along with the separation power of each variable.^2^ The distributions of these variables are compared between the MC predictions and observed data, and found to be well modelled. The BDT discriminant distributions from MC predictions and data are compared and shown in Fig. 4.

**Table 2:** The variables used in the signal region BDT and their separation power (denoted *S*). The variables are derived from the four-momenta of the leading lepton ($\ell _1$), sub-leading lepton ($\ell _2$), the *b*-jet (*b*) and $E_{\text {T}}^{\text {miss}}$ . The last row gives the separation power of the BDT discriminant response

Variable	*S* [$10^{-2}$]
$p_{\text {T}} (\ell _{1} \ell _{2} E_{\text {T}}^{\text {miss}} b)$	4.1
$\Delta p_{\text {T}} (\ell _{1} \ell _{2} b, E_{\text {T}}^{\text {miss}})$	2.5
$\sum {E_{\text {T}}}$	2.3
$\eta (\ell _{1} \ell _{2} E_{\text {T}}^{\text {miss}} b)$	1.3
$\Delta p_{\text {T}} (\ell _{1} \ell _{2}, E_{\text {T}}^{\text {miss}})$	1.1
$p_{\text {T}} (\ell _{1} \ell _{2} b)$	1.0
$C (\ell _{1} \ell _{2})$	0.9
$m (\ell _{2}, b)$	0.2
$m (\ell _{1}, b)$	0.1
BDT response	8.1

To select a signal-enriched portion of events in the signal region, the BDT response is required to be larger than 0.3. The effect of this requirement on event yields is shown in Table 1. The BDT requirement lowers systematic uncertainties by reducing contributions from the $t\bar{t}$ background, which is subject to large modeling uncertainties. For example, the total systematic uncertainty in the fiducial cross-section is reduced by 16% of the total when applying the BDT response requirement, compared to having no requirement. The exact value of the requirement is optimised to reduce the total uncertainty of the measurement over all bins, considering both statistical and systematic uncertainties.

## Unfolding and cross-section determination

The iterative Bayesian unfolding technique in Ref. [14], as implemented in the RooUnfold software package [67], is used to correct for detector acceptance and resolution effects and the efficiency to pass the event selection. The unfolding procedure includes bin-by-bin correction for out-of-fiducial ($C_j^\text {oof}$) events which are reconstructed but fall outside the fiducial acceptance at particle level:

$$ \begin{aligned} C_j^{\text {oof}} = \frac{N^{\mathrm{fid} \& \mathrm{reco}}}{N^\text {reco}}, \end{aligned}$$

followed by the iterative matrix unfolding procedure. The matrix *M* is the migration matrix, and $M^{-1}$ represents the application of the iterative unfolding procedure with migration information from *M*. The iterative unfolding is followed by another bin-by-bin correction to the efficiency to reconstruct a fiducial event ($C_i^{\text {eff}}$):

$$ \begin{aligned} \frac{1}{C_i^{\text {eff}}} = \frac{N^\text {fid}}{N^{\mathrm{fid} \& \mathrm{reco}}}. \end{aligned}$$

In both expressions, “fid” refers to events passing the fiducial selection, “reco” refers to events passing reconstruction-level requirements, and “fid&reco” refers to events passing both. This full unfolding procedure is then described by the expression for the number of unfolded events in bin *i* ($N_i^{\text {ufd}}$) of the particle-level distribution:

$$\begin{aligned} N_i^{\text {ufd}} = \frac{1}{C_i^{\text {eff}}} \sum _j M_{ij}^{-1} C_j^{\text {oof}} \left( N_j^{\text {data}} - B_j\right) , \end{aligned}$$

where *i* (*j*) indicates the bin at particle (reconstruction) level, $N_j^{\text {data}}$ is the number of events in data and $B_j$ is the sum of all background contributions. Table 3 gives the number of iterations used for each observable in this unfolding step. The bias is defined as the difference between the unfolded and true values. The number of iterations is chosen to minimise the growth of the statistical uncertainty propagated through the unfolding procedure while operating in a regime where the bias is sufficiently independent of the number of iterations. The optimal number of iterations is small for most observables, but a larger number is picked for $E(b)$, where larger off-diagonal elements of the migration matrix cause slower convergence of the method.

The list of observables chosen was also checked for shaping induced by the requirement on the BDT response, since strong shaping can make the unfolding unstable. These shaping effects were found to be consistently well-described by the various MC models considered. Any residual differences in the predictions of different MC event generators would increase MC modelling uncertainties, thus ensuring shaping effects of the BDT are covered by the total uncertainties.

**Table 3:** Number of iterations chosen in the unfolding procedure for each of the observables used in the measurement

Observable	Number of iterations
$E(b)$	15
$m(\ell _1 b)$	7
$m(\ell _2 b)$	5
$E(\ell \ell b)$	5
$m_{\text {T}} (\ell \ell \nu \nu b)$	7
$m(\ell \ell b)$	5

Unfolded event yields $N_i^\text {ufd}$ are converted to cross-section values as a function of an observable *X* using the expression:

$$\begin{aligned} \frac{\text {d}\sigma _i}{\text {d}X} = \frac{N_i^{\text {ufd}}}{L \Delta _i}, \end{aligned}$$

where *L* is the integrated luminosity of the data sample and $\Delta _i$ is the width of bin *i* of the particle-level distribution. Differential cross-sections are divided by the fiducial cross-section to create a normalised distribution. The fiducial cross-section is simply the sum of the cross-sections in each bin multiplied by the corresponding bin widths:

$$\begin{aligned} \sigma ^{\text {fid}} = \sum _{i} \left( \frac{\text {d}\sigma _i}{\text {d}X} \cdot \Delta _i \right) = \sum _{i} \frac{N_i^{\text {ufd}}}{L}. \end{aligned}$$

## Systematic uncertainties

### Sources of systematic uncertainty

The experimental sources of uncertainty include the uncertainty in the lepton efficiency scale factors used to correct simulation to data, the lepton energy scale and resolution, the $E_{\text {T}}^{\text {miss}}$ soft-term calculation, the jet energy scale and resolution, the $b\text {-tagging}$ efficiency, and the luminosity.

The JES uncertainty [57] is divided into 18 components, which are derived using $\sqrt{s}={13}\hbox { TeV}$ data. The uncertainties from data-driven calibration studies of $Z/\gamma +$jet and dijet events are represented with six orthogonal components using the eigenvector decomposition procedure, as demonstrated in Ref. [68]. Other components include model uncertainties (such as flavour composition, $\eta$ intercalibration model). The most significant JES uncertainty components for this measurement are the data-driven calibration and the flavour composition uncertainty, which is the dependence of the jet calibration on the fraction of quark or gluon jets in data. The jet energy resolution uncertainty estimate [57] is based on comparisons of simulation and data using studies of Run-1 data. These studies are then cross-calibrated and checked to confirm good agreement with Run-2 data.

As discussed in Sect. 4, the $E_{\text {T}}^{\text {miss}}$ calculation includes contributions from leptons and jets in addition to soft terms which arise primarily from low-$p_{\text {T}}$ pile-up jets and underlying-event activity [62, 63]. The uncertainty associated with the leptons and jets is propagated from the corresponding uncertainties in the energy/momentum scales and resolutions, and it is classified together with the uncertainty associated with the corresponding objects. The uncertainty associated with the soft term is estimated by comparing the simulated soft-jet energy scale and resolution to that in data.

Uncertainties in the scale factors used to correct the $b\text {-tagging}$ efficiency in simulation to the efficiency in data are assessed using independent eigenvectors for the efficiency of *b*-jets, *c*-jets, light-parton jets, and the extrapolation uncertainty for high-$p_{\text {T}}$ jets [59, 60].

Systematic uncertainties in lepton momentum resolution and scale, trigger efficiency, isolation efficiency, and identification efficiency are also considered [52–54]. These uncertainties arise from corrections to simulation based on studies of $Z \rightarrow ee$ and $Z \rightarrow \mu \mu$ data. In this measurement, the effects of the uncertainties in these corrections are relatively small.

A 2.1% uncertainty is assigned to the integrated luminosity. It is derived, following a methodology similar to that detailed in Ref. [69], from a calibration of the luminosity scale using *x*–*y* beam-separation scans.

Uncertainties stemming from theoretical models are estimated by comparing a set of predicted distributions produced with different assumptions. The main uncertainties are due to the NLO matrix-element (ME) event generator, parton shower and hadronisation event generator, radiation tuning and scale choice and the PDF. The NLO matrix-element uncertainty is estimated by comparing two NLO matching methods: the predictions of Powheg-Box and MadGraph5_aMC@NLO, both interfaced with Herwig++. The parton shower, hadronisation, and underlying-event model uncertainty is estimated by comparing Powheg-Box interfaced with either Pythia 6 or Herwig++. The uncertainty from the matrix-element event generator is treated as uncorrelated between the *tW* and $t\bar{t}$ processes, while the uncertainty from the parton shower event generator is treated as correlated. The radiation tuning and scale choice uncertainty is estimated by taking half of the difference between samples with Powheg-Box interfaced with Pythia 6 tuned with either more or less radiation, and is uncorrelated between the *tW* and $t\bar{t}$ processes. These choices of correlations are based on Ref. [11], and were checked to be no less conservative than the alternative options. The choice of scheme to account for the interference between the *tW* and $t\bar{t}$ processes constitutes another source of systematic uncertainty for the signal modelling, and it is estimated by comparing samples using either the diagram removal scheme or the diagram subtraction scheme, both generated with Powheg-Box +Pythia 6. The uncertainty due to the choice of PDF is estimated using the PDF4LHC15 combined PDF set [70]. The difference between the central CT10 [31] prediction and the central PDF4LHC15 prediction (PDF central value) is taken and symmetrised together with the internal uncertainty set provided with PDF4LHC15.

Additional normalisation uncertainties are applied to each background. A 100% uncertainty is applied to the normalisation of the background from non-prompt and fake leptons, an uncertainty of 50% is applied to the $Z {\,\text {+}\,\text {jets}}$ background, and a 25% normalisation uncertainty is assigned to diboson backgrounds. These uncertainties are based on earlier ATLAS studies of background simulation in top quark analyses [71]. These normalisation uncertainties are not found to have a large impact on the final measurement due to the small contribution of these backgrounds in the signal region as well as their cancellation in the normalised cross-section measurement. An uncertainty of 5.5% is applied to the $t\bar{t}$ normalisation to account for the scale, $\alpha _{\text {S}}$, and PDF uncertainties in the NNLO cross-section calculation.

Uncertainties due to the size of the MC samples are estimated using pseudoexperiments. An ensemble of pseudodata is created by fluctuating the MC samples within the statistical uncertainties. Each set of pseudodata is used to construct $M_{ij}$, $C_i^\text {eff}$, and $C_j^\text {oof}$, and the nominal MC sample is unfolded. The width of the distribution of unfolded values from this ensemble is taken as the statistical uncertainty. Additional non-closure uncertainties are added in certain cases after stress-testing the unfolding procedure with injected Gaussian or linear functions. Each distribution is tested by reweighting the input MC sample according to the injected function, unfolding, and checking that the weights are recovered in the unfolded distribution. The extent to which the unfolded weighted data are biased with respect to the underlying weighted generator-level distribution is taken as the unfolding non-closure uncertainty.

### Procedure for estimation of uncertainty

The propagation of uncertainties through the unfolding process proceeds by constructing the migration matrix and efficiency corrections with the baseline sample and unfolding with the varied sample as input. In most cases, the baseline sample is from Powheg-Box +Pythia 6 and produced with the full detector simulation, but in cases where the varied sample uses the Atlfast2 fast simulation, the baseline sample is also changed to use Atlfast2. For uncertainties modifying background processes, varied samples are prepared by taking into account the changes in the background induced by a particular systematic effect. Experimental uncertainties are treated as correlated between signal and background in this procedure. The varied samples are unfolded and compared to the corresponding particle-level distribution from the MC event generator; the relative difference in each bin is the estimated systematic uncertainty.

The covariance matrix $\mathbf {C}$ for each differential cross-section measurement is computed following a procedure similar to that used in Ref. [72]. Two covariance matrices are summed to form the final covariance. The first one is computed using 10,000 pseudoexperiments and includes statistical uncertainties as well as systematic uncertainties from experimental sources. The statistical uncertainties are included by independently fluctuating each bin of the data distribution according to Poisson distributions for each pseudoexperiment. Each bin of the resulting pseudodata distribution is then fluctuated according to a Gaussian distribution for each experimental uncertainty, preserving bin-to-bin correlation information for each uncertainty. The other matrix includes the systematic uncertainties from event generator model uncertainties, PDF uncertainties, unfolding non-closure uncertainties, and MC statistical uncertainties. In this second matrix, the bin-to-bin correlation value is set to zero for the non-closure and MC statistical uncertainties, and set to unity for the other uncertainties. The impact of setting the bin-to-bin correlation value to unity was compared for the non-closure uncertainty, and this choice was found to have negligible impact on the results. This covariance matrix is used to compute a $\chi ^2$ and corresponding *p* value to assess how well the measurements agree with the predictions. The $\chi ^2$ values are computed using the expression:

$$\begin{aligned} \chi ^2 = \mathbf {v}^\intercal \mathbf {C}^{-1} \mathbf {v}, \end{aligned}$$

where $\mathbf {v}$ is the vector of differences between the measured cross-sections and predictions.

## Results

**Fig. 5:**
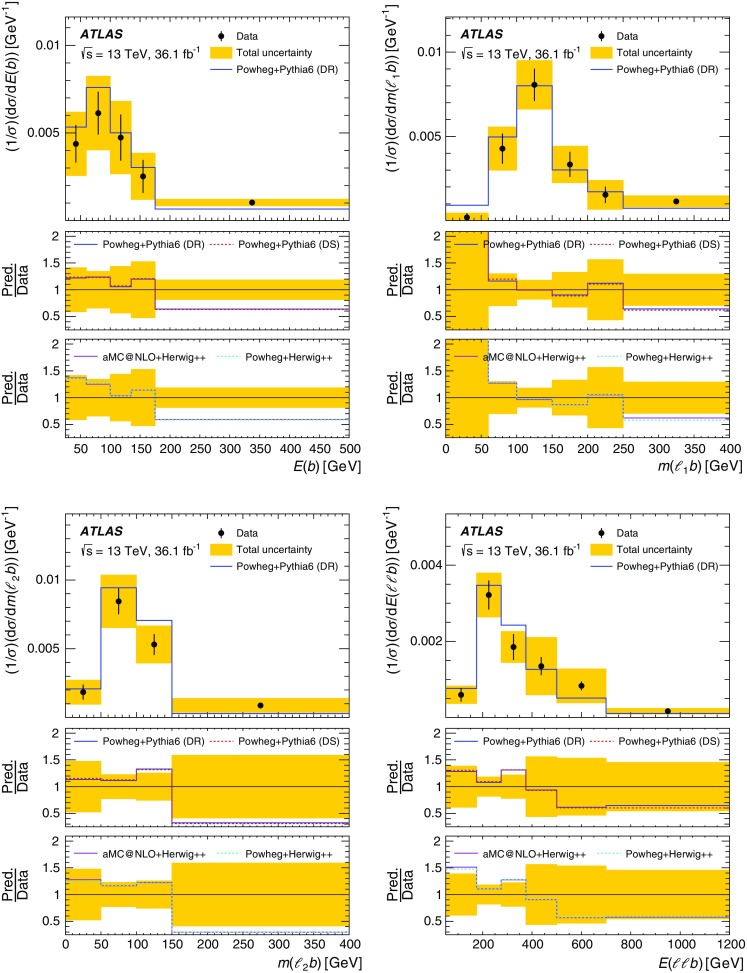
Normalised differential cross-sections unfolded from data, compared with selected MC models, with respect to $E(b)$, $m(\ell _1 b)$, $m(\ell _2 b)$, and $E(\ell \ell b)$. Data points are placed at the horizontal centre of each bin, and the error bars on the data points show the statistical uncertainties. The total uncertainty in the first bin of the $m(\ell _1 b)$ distribution (not shown) is 140%. See Sect. 1 for a description of the observables plotted

**Fig. 6:**
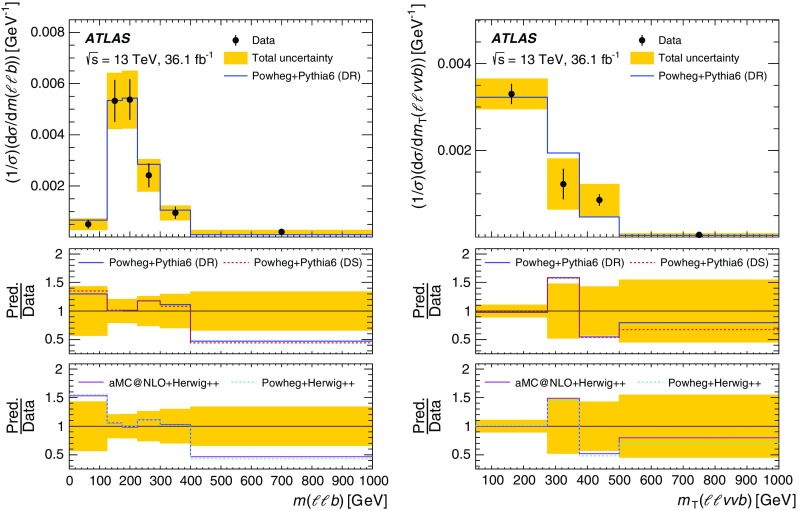
Normalised differential cross-sections unfolded from data, compared with selected MC models, with respect to $m_{\text {T}} (\ell \ell \nu \nu b)$ and $m(\ell \ell b)$. Data points are placed at the horizontal centre of each bin. See Sect. 1 for a description of the observables plotted

**Fig. 7:**
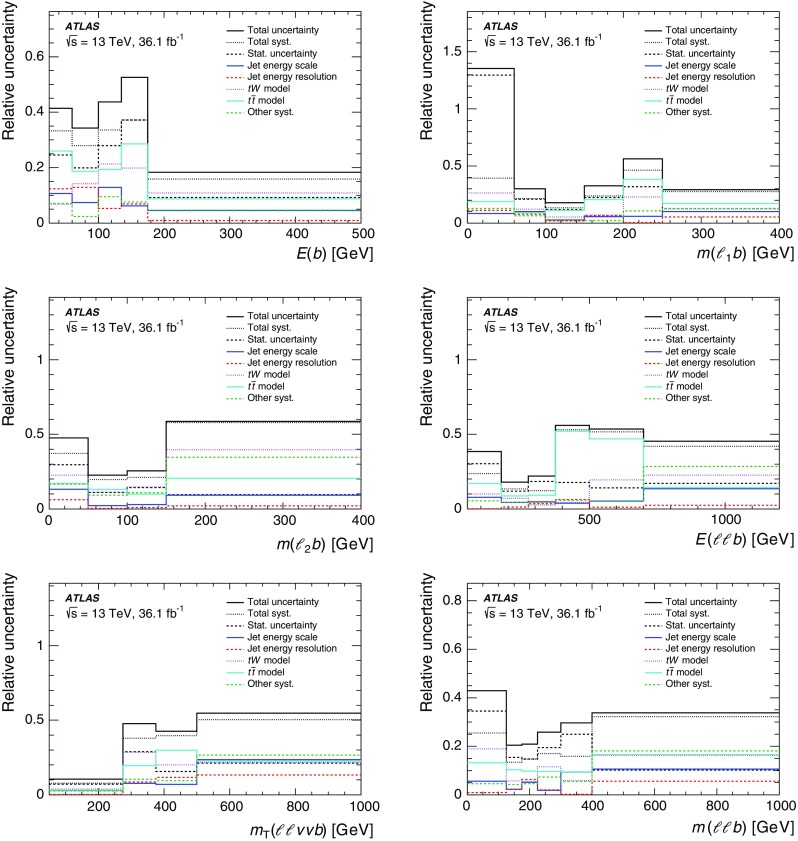
Summary of uncertainties in normalised differential cross-sections unfolded from data

Unfolded particle-level normalised differential cross-sections are given in Table 4. In Figs. 5 and 6, the results are shown compared to the predictions of various MC event generators, and in Fig. 7 the main systematic uncertainties for each distribution are summarised. The results show that the largest uncertainties come from the size of the data sample as well as $t\bar{t}$ and *tW* MC modelling.

The comparison between the data and Monte Carlo predictions is summarised in Table 5, where $\chi ^2$ values and corresponding *p* values are listed. In general, most of the MC models show fair agreement with the measured cross-sections, with no particularly low *p* values observed. Notably, for each distribution there is a substantial negative slope in the ratio of predicted to observed cross-sections, indicating there are more events with high-momentum final-state objects than several of the MC models predict. This effect is most visible in the $E(\ell \ell b)$ distribution, where the lower *p* values for all MC predictions reflect this. In most cases, differences between the MC predictions are smaller than the uncertainty on the data, but there are some signs that Powheg-Box +Herwig++ deviates more from the data and from the other predictions in certain bins of the $E(\ell \ell b)$ , $m(\ell \ell b)$ , and $m(\ell _1 b)$ distributions. The predictions of DS and DR samples likewise give very similar results for all observables as expected from the fiducial selection. The predictions of Powheg-Box +Pythia 6 with varied initial- and final-state radiation tuning were also examined but not found to give significantly different distributions in the fiducial phase space of this analysis.

Both the statistical and systematic uncertainties have a significant impact on the result. The exact composition varies bin-to-bin but there is no single source of uncertainty that dominates each normalised measurement. Some of the largest systematic uncertainties are those related to $t\bar{t}$ and *tW* modelling. The cancellation in the normalised differential cross-sections is very effective at reducing a number of systematic uncertainties. The most notable cancellation is related to the $t\bar{t}$ parton shower model uncertainty, which is quite dominant prior to dividing by the fiducial cross-section.

**Table 4:** Summary of the measured normalised differential cross-sections, with uncertainties shown as percentages. The uncertainties are divided into statistical and systematic contributions

$E(b)$ bin [GeV]	[25, 60]	[60, 100]	[100, 135]	[135, 175]	[175, 500]	
$(1/\sigma ) \;\text {d}\sigma /\text {d}x$ [$\hbox {GeV}^{-1}$]	0.00438	0.00613	0.00474	0.00252	0.00103	
Stat. uncertainty	25	20	28	37	9.3	
Total syst. uncertainty	33	28	34	37	16	
Total uncertainty	41	34	44	53	18	

$m(\ell _1 b)$ bin [GeV]	[0, 60]	[60, 100]	[100, 150]	[150, 200]	[200, 250]	[250, 400]
$(1/\sigma ) \;\text {d}\sigma /\text {d}x$ [$\hbox {GeV}^{-1}$]	0.000191	0.00428	0.00806	0.00333	0.00153	0.00114
Stat. uncertainty	130	21	12	22	32	10
Total syst. uncertainty	39	22	13	24	46	28
Total uncertainty	140	30	18	33	56	29

$m(\ell _2 b)$ bin [GeV]	[0, 50]	[50, 100]	[100, 150]	[150, 400]		
$(1/\sigma ) \;\text {d}\sigma /\text {d}x$ [$\hbox {GeV}^{-1}$]	0.00184	0.00845	0.00531	0.000879		
Stat. uncertainty	30	11	14	9.6		
Total syst. uncertainty	37	20	21	58		
Total uncertainty	48	23	25	59		

$E(\ell \ell b)$ bin [GeV]	[50, 175]	[175, 275]	[275, 375]	[375, 500]	[500, 700]	[700, 1200]
$(1/\sigma ) \;\text {d}\sigma /\text {d}x$ [$\hbox {GeV}^{-1}$]	0.000597	0.00322	0.00185	0.00135	0.000832	0.000167
Stat. uncertainty	30	12	18	18	14	17
Total syst. uncertainty	24	13	12	53	52	42
Total uncertainty	38	18	22	56	53	45

$m_{\text {T}} (\ell \ell \nu \nu b)$ bin [GeV]	[50, 275]	[275, 375]	[375, 500]	[500, 1000]		
$(1/\sigma ) \;\text {d}\sigma /\text {d}x$ [$\hbox {GeV}^{-1}$]	0.0033	0.00123	0.000856	$5.51\times 10^{-5}$		
Stat. uncertainty	7.1	29	16	21		
Total syst. uncertainty	7.8	38	40	50		
Total uncertainty	11	48	43	55		

$m(\ell \ell b)$ bin [GeV]	[0, 125]	[125, 175]	[175, 225]	[225, 300]	[300, 400]	[400, 1000]
$(1/\sigma ) \;\text {d}\sigma /\text {d}x$ [$\hbox {GeV}^{-1}$]	0.00051	0.00533	0.00538	0.00242	0.000949	0.000208
Stat. uncertainty	35	15	15	19	25	10
Total syst. uncertainty	25	13	15	17	16	32
Total uncertainty	43	20	21	26	30	34

**Table 5:** Values of $\chi ^2$ and *p* values for the measured normalised cross-sections compared to particle-level MC predictions

Observable	$E(b)$		$m(\ell _1 b)$		$m(\ell _2 b)$		$E(\ell \ell b)$		$m_{\text {T}} (\ell \ell \nu \nu b)$		$m(\ell \ell b)$	
Degrees of freedom	4		5		3		5		3		5	
Prediction	$\chi ^2$	*p*	$\chi ^2$	*p*	$\chi ^2$	*p*	$\chi ^2$	*p*	$\chi ^2$	*p*	$\chi ^2$	*p*
Powheg +Pythia 6 (DR)	4.8	0.31	5.7	0.34	2.6	0.45	8.1	0.15	2.0	0.56	4.0	0.55
Powheg +Pythia 6 (DS)	5.0	0.29	6.1	0.30	2.6	0.46	9.1	0.11	2.4	0.49	4.4	0.50
aMC@NLO+Herwig++	5.6	0.23	5.4	0.37	2.4	0.49	8.7	0.12	1.8	0.61	3.6	0.61
Powheg +Herwig++	6.2	0.18	8.1	0.15	2.3	0.52	11.0	0.05	2.0	0.57	5.2	0.40
Powheg +Pythia 6 radHi	4.8	0.30	5.3	0.38	2.5	0.48	7.9	0.16	1.9	0.60	3.7	0.60
Powheg +Pythia 6 radLo	5.0	0.29	5.8	0.33	2.6	0.45	8.4	0.14	2.1	0.56	4.0	0.55

## Conclusion

The differential cross-section for the production of a *W* boson in association with a top quark is measured for several particle-level observables. The measurements are performed using $36.1~\hbox {fb}^{-1}$ of *pp* collision data with $\sqrt{s} =13~\hbox {TeV}$ collected in 2015 and 2016 by the ATLAS detector at the LHC. Cross-sections are measured in a fiducial phase space defined by the presence of two charged leptons and exactly one jet identified as containing *b*-hadrons. Six observables are chosen, constructed from the masses and energies of leptons and jets as well as the transverse momenta of neutrinos. Measurements are normalised with the fiducial cross-section, causing several of the main uncertainties to cancel out. Dominant uncertainties arise from limited data statistics, signal modelling, and $t\bar{t}$ background modelling. Results are found to be in good agreement with predictions from several MC event generators.

## Footnotes

1 ATLAS uses a right-handed coordinate system with its origin at the nominal interaction point (IP) in the centre of the detector and the *z*-axis along the beam pipe. The *x*-axis points from the IP to the centre of the LHC ring, and the *y*-axis points upward. Cylindrical coordinates $(r,\phi )$ are used in the transverse plane, $\phi$ being the azimuthal angle around the *z*-axis. The pseudorapidity is defined in terms of the polar angle $\theta$ as $\eta =-\ln \tan (\theta /2)$, while the rapidity is defined in terms of particle energies and the *z*-component of particle momenta as $y=(1/2)\ln \left[ (E+p_z)/(E-p_z)\right]$.

2 The separation power, *S*, is a measure of the difference between probability distributions of signal and background in the variable, and is defined as:

$$\begin{aligned} \langle S^2 \rangle = \frac{1}{2} \int { \frac{{\left( Y_{\text {s}}(y) - Y_{\text {b}}(y)\right) }^2}{Y_{\text {s}}(y)+Y_{\text {b}}(y)} \text {d}y} \end{aligned}$$

where $Y_{\text {s}}(y)$ and $Y_{\text {b}}(y)$ are the signal and background probability distribution functions of each variable *y*, respectively.
